# DEEPred: Automated Protein Function Prediction with Multi-task Feed-forward Deep Neural Networks

**DOI:** 10.1038/s41598-019-43708-3

**Published:** 2019-05-14

**Authors:** Ahmet Sureyya Rifaioglu, Tunca Doğan, Maria Jesus Martin, Rengul Cetin-Atalay, Volkan Atalay

**Affiliations:** 1 Department of Computer Engineering, METU, Ankara, 06800 Turkey; 2 Department of Computer Engineering, İskenderun Technical University, Hatay, 31200 Turkey; 3 European Molecular Biology Laboratory, European Bioinformatics Institute (EMBL-EBI), Hinxton, Cambridge CB10 1SD UK; 4 KanSiL, Department of Health Informatics, Graduate School of Informatics, METU, Ankara, 06800 Turkey

**Keywords:** Data mining, Gene ontology, Machine learning, Protein function predictions, Sequence annotation

## Abstract

Automated protein function prediction is critical for the annotation of uncharacterized protein sequences, where accurate prediction methods are still required. Recently, deep learning based methods have outperformed conventional algorithms in computer vision and natural language processing due to the prevention of overfitting and efficient training. Here, we propose DEEPred, a hierarchical stack of multi-task feed-forward deep neural networks, as a solution to Gene Ontology (GO) based protein function prediction. DEEPred was optimized through rigorous hyper-parameter tests, and benchmarked using three types of protein descriptors, training datasets with varying sizes and GO terms form different levels. Furthermore, in order to explore how training with larger but potentially noisy data would change the performance, electronically made GO annotations were also included in the training process. The overall predictive performance of DEEPred was assessed using CAFA2 and CAFA3 challenge datasets, in comparison with the state-of-the-art protein function prediction methods. Finally, we evaluated selected novel annotations produced by DEEPred with a literature-based case study considering the ‘biofilm formation process’ in *Pseudomonas aeruginosa*. This study reports that deep learning algorithms have significant potential in protein function prediction; particularly when the source data is large. The neural network architecture of DEEPred can also be applied to the prediction of the other types of ontological associations. The source code and all datasets used in this study are available at: https://github.com/cansyl/DEEPred.

## Introduction

Functional annotation of proteins is crucial for understanding the cellular mechanisms, identifying disease-causing functional changes in genes/proteins, and for discovering novel tools for disease prevention, diagnosis, and treatment. Traditionally, gene/protein functions are first identified by *in vitro* and *in vivo* experiments and recorded in biological databases via literature-based curation. However, wet-lab experiments and manual curation efforts are cumbersome and time consuming. Thus, they are unable to resolve the knowledge gap that is being produced due to the continuous growth of biological sequence data^1^. Therefore, accurate computational methods have been sought to automatically annotate functions of proteins.

The Gene Ontology (GO) provides a controlled vocabulary to classify the attributes of proteins based upon representative terms, referred to as “GO terms”^2^. The GO system divides protein attributes into three main categories: molecular function (MF), biological process (BP) and cellular component (CC). Each GO term represents a unique functional attribute and all terms are associated to each other in a directed acyclic graph (DAG) structure based on inheritance relationships. Several GO term-based protein function prediction methods have been proposed in the last decade to automatically annotate protein sequences using machine learning and statistical analysis techniques^3–8^. Considering the prediction performances of the current methods, it can be stated that there is still room for significant improvement in this area. Critical Assessment of Protein Function Annotation (CAFA) is an initiative, whose aim is the large-scale evaluation of protein function prediction methods, and the results of the first two CAFA challenges showed that protein function prediction is still a challenging area^9,10^.

Several machine learning techniques have been employed for protein function prediction, such as the artificial neural networks (ANNs)^11^. Deep Neural Network (DNN) algorithms, a sub-group of ANNs, have multiple hidden layers. DNNs take low level features as input and build more advanced features at each subsequent layer. DNN-based methods have already become industry standards in the fields of computer vision and natural language processing^12–16^. Recent improvements in affordable computational power have allowed the scientific community to apply DNN-based methods on numerous research fields including biomedical data analysis; where, DNN algorithms have been shown to outperform the traditional predictive methods in bioinformatics and cheminformatics^17–21^. DNNs are divided into two groups in terms of the task modelling approach. Multi-task DNNs are designed for classifying the input instances into multiple pre-defined classes/tasks^22^, as opposed to single-task DNNs, where the aim is to make a binary prediction. In terms of the model architecture and properties, DNNs are classified into multiple groups, the most popular architectures are feed-forward DNN (i.e., multi-layered perceptron), recurrent neural network (RNN), restricted Boltzmann machine (RBM) and deep belief network (DBN), auto encoder deep neural networks, convolutional neural network (CNN), and graph convolutional network (GCN)^14,15,18,19,22,23^.

Investigative studies showed that, applications of multi-task DNNs provided a significant performance increase in ligand-based drug discovery. Ligand-based drug discovery can be considered similar to the problem of protein function prediction^21,24^. In protein function prediction, the associations between the ontology-based function defining terms (e.g., GO terms) and proteins are identified, where a protein may have more than one functional association. Therefore, protein function prediction is a multi-label learning problem and thus can be solved using multi-task deep neural networks, similar to the applications in drug discovery^25^. Multi-task DNN algorithms inherently extract the relationships between multiple classes by building complex features from the raw input data at each layer in a hierarchical manner. Additionally, shared hidden units among different classes enhance the prediction results of the classes that have a low number of training samples, which often has a positive impact on the predictive performance.

To the best of our knowledge, deep learning algorithms have not been thoroughly investigated in terms of generating practical large-scale protein function prediction pipelines. However, there have been a some studies mostly confined to small sets of proteins and functional classes. In these studies, DNNs were applied to predict protein functions using different types of protein features such as amino acid sequences^26–29^, 3-D structural properties^30^, protein-protein interaction networks^28,31^ or other molecular and functional aspects^29,32–34^, and various types of DNN architectures such as single or multi-task feed-forward DNNs^32^, recurrent neural networks^26,27^, deep autoencoder neural networks^31,33^, deep restricted Boltzmann machines^34^ or convolutional neural networks^28–30^. We have discussed and compared each study mentioned above in the Supplementary Material 1 Document, Section 1.

One of the most critical obstacles against developing a practical DNN-based predictive tool is the computationally intensive training processes that limits the size of input data and the number of functional categories that can be included in the system. Due to this reason, previous studies mostly focused on a small number of protein families or GO terms. Whilst, methods covering large sets of GO terms suffered from long training duration and reduced predictive performance issues. Therefore, there is a need for new predictive approaches not only with high performance, but also with real-world usability, to be able to support *in vitro* studies in protein function identification.

In this study, we propose a novel multi-task hierarchical deep learning method, DEEPred, for the prediction of GO term associations to protein sequence records in biological data resources such as the UniProtKB, as well as for poorly and uncharacterized open reading frames. We also provide a comprehensive investigation on DNN-based predictive model characteristics when applied on protein sequence and ontology data. Our initial preprint work on this topic was one of the first applications of deep neural networks for sequence based protein function prediction^35^. This study contributes to the existing literature in terms of designing a large-scale deep learning based predictive system using a stack of 1,101 multi-task feed-forward DNNs, capable of predicting thousands of Gene Ontology based functional definitions. Additionally, the prediction of GO terms with very low number of training instances, which is a major problem in the field of automated protein function prediction, has been addressed by proposing a practical data augmentation solution by incorporating previously produced automated functional predictions into the system training.

## Results

The technical details of DEEPred are given in the Methods section. The results of several performance and validation analyses are provided below.

### Input feature type performance analysis

In predictive modeling, input instances/samples are quantized as feature vectors, and these feature vectors are required to reflect the intrinsic properties of the samples they represent, which should also be correlated with their known labels (i.e., GO term associations in our case). For this reason, finding the best representative feature type is important for any machine learning application. In this analysis, our aim was to investigate the best representative feature type for proteins, to be incorporated in DEEPred. For this purpose, we randomly selected three DEEPred DNN models that contain MF GO terms from different levels on the GO hierarchy, and trained each model using three different feature types (i.e., SPMap, pseudo amino acid composition - PAAC and the conjoint triad) as explained in the Methods section. The reason behind using MF GO term models was MF being the most clearly defined aspect of GO and also the easiest one to predict.

We measured the performance of the models using cross-validation, with 80% to 20% separation of the source training data, to observe the best representative feature. Table 1 shows the selected models together with the incorporated GO terms, their levels on the GO DAG, the number of annotated proteins and the performances for each feature type. The average performances (F1-score) were calculated as 0.63, 0.36 and 0.43 for SPMap, PAAC and the conjoint triad features, respectively. Since the predictive performance with SPMap feature was the best, we incorporated SPMap into the DEEPred system for the rest of the study.

**Table 1:** Input feature type performance comparison results.

Model & GO level	GO term id	GO description	# of annotated proteins	Predictive performance (F1-score)		
				SPMap	Pseudo-amino acid composition	Conjoint triad
Model 1 (GO level: 2)	GO:0036094	small molecule binding	1 847	0.49	0.29	0.23
	GO:0003700	DNA binding transcription factor activity	1 652			
	GO:0004872	receptor activity	1 332			
	GO:0044877	protein-containing complex binding	1 296			
	GO:0097367	carbohydrate derivative binding	1 252			
Model 2 (GO level: 4)	GO:0004529	exodeoxyribonuclease activity	50	0.68	0.53	0.38
	GO:0045309	protein phosphorylated amino acid binding	50			
	GO:0008395	steroid hydroxylase activity	49			
	GO:0008649	rRNA methyltransferase activity	49			
	GO:0015645	fatty acid ligase activity	49			
Model 3 (GO level: 7)	GO:0001012	RNA polymerase II regulatory region DNA binding	818	0.74	0.53	0.47
	GO:0016887	ATPase activity	764			
	GO:0046873	metal ion transmembrane transporter activity	685			
	GO:0001159	core promoter proximal region DNA binding	504			
	GO:0015077	monovalent inorganic cation transmembrane transporter activity	480			

### Effect of training dataset sizes on system performance

DNN models usually require a high number of training instances in order to produce accurate predictions. A significant disadvantage in this regard is that, large-scale biological training datasets are not generally available. One solution to this problem would be to discard GO terms with a low number of training instances from the system. In this case, the problem is that there are only a small number of GO terms available for prediction, most of which are shallow (i.e., generic and less informative terms). In order to investigate the effect of training dataset sizes on the predictive performance, we carried out a detailed analysis with multiple training and testing processes.

**Figure 1:**
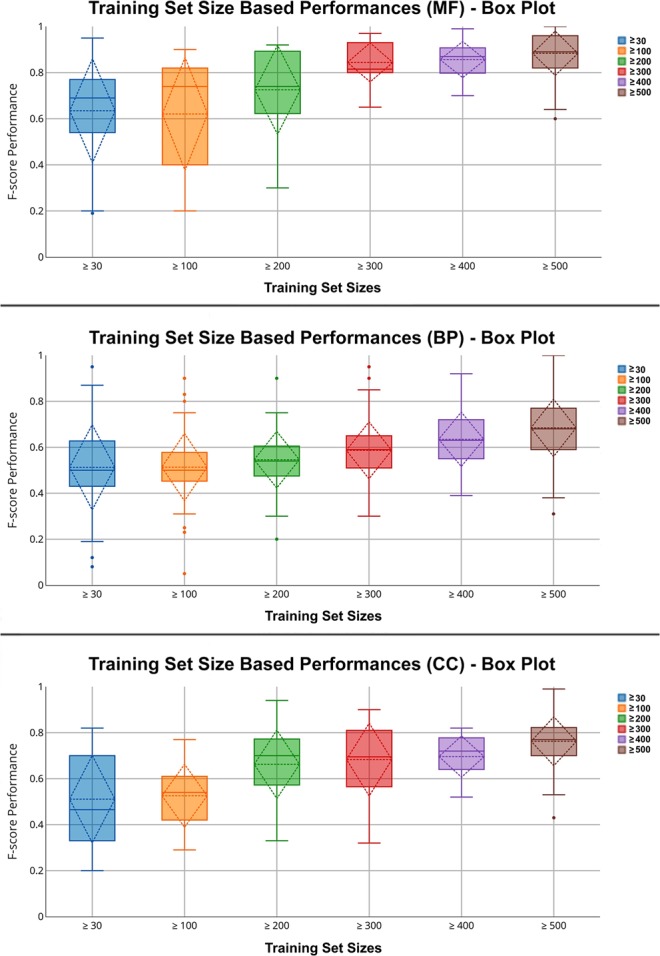
Box plots for training dataset size specific performance evaluation. Each box plot represents variance, mean and standard deviations of F1-score values (vertical axis) for models with differently sized training datasets (horizontal axis), for each GO category. In this analysis, the training was done using only the annotations with manual and experimental evidence codes.

We constructed 6 different training datasets based on the annotated protein counts of different GO terms, as described in the Methods section. Table 2 summarizes the training dataset sizes and contents based upon the MF annotations. There are two vertical blocks in Table 2, the first one belongs to “Annotations with only manual experimental evidence codes”; and the second block belongs to “Annotations with all evidence codes”. As observed from the first block, the number of available GO levels and GO terms decreases as the minimum compulsory number of annotations increases, since specific GO terms usually have less number of annotations. We trained the DEEPred system with each of these training datasets (i.e., annotations with only manual experimental evidence codes) and measured the predictive performance individually. We then compared them with each other to observe if there is a correlation. The average performance of the models for each training dataset is given in Table 3 and in Fig. 1. Each column in Table 3 corresponds to an average F1-score value of the GO terms belonging to a particular training dataset. Box plots in Fig. 1 additionally display median and variance values. Here, it is evident that there is a strong correlation between the training sample size and performance. As expected, increasing the training dataset sizes elevated the classification performance for all GO categories. High variance values at low training dataset sizes indicates that these models are less stable. In this part of the study, we also carried out a GO level specific performance analysis. The details of this analysis can be found in the Supplementary Material 1 Document, Section 7.

**Table 2:** Statistics for the training datasets created by only using annotations with manual and experimental evidence codes and the training datasets created by using annotations with all evidence codes.

Training Dataset Statistics							
	Annotation Count	Annotations with only manual experimental evidence codes			Annotations with all evidence codes		
		# of available levels	# of GO terms	# of annotations	# of available levels	# of GO terms	# of annotations
Molecular Function	**≥30**	9	838	281 125	11	2 776	6 451 530
	**≥100**	9	605	272 235	10	1 598	6 386 105
	**≥200**	9	395	257 404	10	1 174	6 326 109
	**≥300**	8	226	233 476	9	942	6 269 643
	**≥400**	8	165	218 591	9	809	6 223 762
	**≥500**	8	142	210 790	9	698	6 173 867
Biological Process	**≥30**	10	4 215	1 433 220	12	8 404	16 537 812
	**≥100**	10	2 993	1 386 588	12	4 768	16 335 538
	**≥200**	9	1 782	1 302 577	11	3 299	16 129 271
	**≥300**	9	1 059	1 199 604	10	2 631	15 965 583
	**≥400**	8	743	1 123 037	9	2 233	15 828 012
	**≥500**	8	603	1 075 353	9	1 978	15 713 431
Cellular Comp.	**≥30**	7	606	340 995	8	1 268	4 167 000
	**≥100**	6	460	335 445	8	750	4 138 327
	**≥200**	6	324	325 687	7	549	4 110 383
	**≥300**	6	206	309 390	6	442	4 083 834
	**≥400**	6	155	296 929	6	377	4 061 654
	**≥ 500**	5	118	283 616	6	335	4 043 150

**Table 3:** The average prediction performance (F1-score) for GO term models belonging to different training dataset size bins.

GO categories	Performance measures (F1-score) for different training dataset sizes					
	≥ 30	≥ 100	≥ 200	≥ 300	≥ 400	≥ 500
Molecular Function	0.66	0.68	0.77	0.82	0.82	0.83
Biological Process	0.42	0.50	0.52	0.52	0.56	0.55
Cellular Component	0.50	0.59	0.64	0.63	0.64	0.65

In this analysis, the training was done using only the annotations with manual and experimental evidence codes.

### Performance evaluation of training with electronic annotations

In DEEPred, the minimum required number of annotated proteins for each GO term (to be used in the training) is 30, which was considered as the minimum number required for statistical power. Due to this threshold, all GO terms with less than 30 annotated proteins were eliminated from the system. The eliminated terms corresponded to 25,257 out of 31,352 GO terms (31,352 is the total number of terms that have been annotated to at least one UniProtKB/Swiss-Prot protein entry with manual and experimental evidence codes), which can be considered as a significant loss. The same problem exists for most of the machine learning based methods in the automated protein function prediction domain. Moreover, the DNN models with a low or moderate number of training instances (i.e., between 30 to 100 for each incorporated GO term) displayed lower performance compared to the models with high number of training samples, as discussed above. In this section, we investigated a potential way to increase the statistical power of our models by enriching the training datasets.

In the UniProtKB/SwissProt database, only 1% of the total number of GO term annotations are tagged with manual and experimental evidence codes. The remaining of the GO term annotations are electronically made (evidence code: IEA), and these annotations are usually considered as less reliable due to potential errors (i.e., false positives). Normally, electronic annotations are not used for system training to avoid error propagation. In this test, we investigated the performance change when all annotations (including electronic ones) were included in the training procedure of DEEPred, and we discussed whether deep learning algorithms could handle noisy training data, as stated in the literature.

To perform this experiment, we first identified the MF GO terms whose annotation count was increased at least four times, when electronically made annotations were incorporated. We randomly selected 25 MF GO terms that satisfied this condition, and trained/evaluated the models with 80% to 20% training-validation separation, similar to our previous tests. The training dataset sizes and performance values for the “all-annotation-training” analysis are given in Table 4. In this table, we divided GO terms into two main categories as “previously high performance models” and “previously low performance models” based on the performances when the system was trained only with annotations of manual experimental evidence codes. The results showed that adding electronic annotations to the training procedure increased the performances of selected “previously low performance models”. On the other hand, including electronic annotations in the training of “previously high performance models” decreased their performances in some of the cases. Overall, the performance change was positive.

**Table 4:** Performance (F1-score) changes for the selected GO terms after the enrichment of training datasets with electronic annotations. In this analysis, the training was done using all of the available annotations, without any selection based on the evidence code.

	GO Term	GO Description	NoA* (ME*)	NoA (AE*)	F1-score perf. (ME)	F1-score perf. (AE)	Perf. Change
Previously low performance models	GO:0070569	uridylyltransferase activity	35	970	0.58	0.88	0.30
	GO:0019203	carbohydrate phosphatase activity	63	681	0.51	0.84	0.33
	GO:0004197	cysteine-type endopeptidase activity	100	853	0.45	0.40	-0.05
	GO:0005524	ATP binding	596	85 442	0.53	0.93	0.40
	GO:0030554	adenyl nucleotide binding	689	86 319	0.51	0.90	0.39
	GO:0035639	purine ribonucleoside triphosphate binding	834	98 924	0.43	0.80	0.37
	GO:0032555	purine ribonucleotide binding	951	99 286	0.51	0.89	0.38
	GO:0097367	carbohydrate derivative binding	1 395	10 413	0.41	0.73	0.32
	GO:0000166	nucleotide binding	1 487	116 408	0,53	0.82	0.29
	GO:0036094	small molecule binding	2 059	12 634	0.40	0.72	032
	GO:0043169	cation binding	2 145	119 698	0.48	0.71	0.23
	GO:0043167	ion binding	4 132	20 278	0.33	0.79	0.46
Previously high performance models	GO:0004784	superoxide dismutase activity	38	459	0.81	0.68	-0.13
	GO:0004004	ATP-dependent RNA helicase activity	48	954	0.75	0.73	-0.02
	GO:0005525	GTP binding	258	14 479	0.95	0.79	-0.16
	GO:0032550	purine ribonucleoside binding	286	14 496	0.89	0.62	-0.27
	GO:0001883	purine nucleoside binding	289	14 506	0.89	0.87	-0.02
	GO:0032549	ribonucleoside binding	296	15 460	0.78	0.80	0.02
	GO:0001882	nucleoside binding	304	15 508	0.92	0.79	-0.13
	GO:0008270	zinc ion binding	520	11 385	0.83	0.71	-0.12
	GO:0032559	adenyl ribonucleotide binding	673	85 691	0.80	0.80	0.00
	GO:0017076	purine nucleotide binding	975	99 924	0.91	0.65	-0.26
	GO:0032553	ribonucleotide binding	1 025	100 844	0.87	0.61	-0.26
	GO:0046872	metal ion binding	1 985	118 577	0.81	0.80	-0.01
	GO:0004784	superoxide dismutase activity	38	459	0.81	0.68	-0.13
	**Average**		**861**	**47 658**	**0.68**	**0.77**	**0.10**

*NoA: Number of Annotations, ME: Manual-Experimental Evidence, AE: All Evidence.

### Evaluation of the overall system performance

For the final system training of DEEPred, we used the training dataset of GO terms with at least 30 annotated proteins (with manual and experimental evidence codes); this dataset was also used to measure the overall performance of DEEPred.

The overall system performance was evaluated by considering all 1,101 predictive models. For testing, the hold-out dataset (see Methods) was employed, which was not used at all during system training. The test proteins were fed to all of the models and the system performance was calculated using precision, recall and F1-score (Table 5). The average prediction performance (F1-score) was calculated as 0.62, 0.46 and 0.55 for MF, BP and CC categories, respectively, without using the hierarchical post-processing method (see Methods). When we employed the hierarchical post-processing procedure, which represents the finalized version of DEEPred, the overall average system performance (F1-score) was increased to 0.67, 0.51 and 0.58 for MF, BP and CC categories respectively.

**Table 5:** The average overall performance results of DEEPred, with and without the hierarchical post-processing procedure.

	without Hierarchical Post-processing			with Hierarchical Post-processing		
	F1-score	Precision	Recall	F1-score	Precision	Recall
Molecular Function	0.62	0.52	0.77	0.67	0.61	0.74
Biological Process	0.46	0.36	0.65	0.51	0.44	0.62
Cellular Component	0.55	0.50	0.61	0.58	0.58	0.58

In this analysis, the training was done using the annotations with manual and experimental evidence codes.

### Performance comparison against the state-of-the-art

Two separate analyses were carried out for the comparison against the state-of-the-art. In the first one, the CAFA2 challenge data was used. In CAFA2, GO term based function predictions of 126 methods from 56 research groups were evaluated. The performance results of best performing 10 methods are available in the CAFA2 report^9^. In order to yield a fair comparison with the CAFA2 participating methods, DEEPred models were re-trained using the GO annotation data from September 2013. Afterwards, DEEPred was run on the CAFA2 benchmark protein sequences and the performance results (F-max) of 0.49, 0.26, and 0.43 were obtained for MF, BP, and CC categories respectively; considering the no-knowledge benchmark set in the full evaluation mode (the official CAFA2 performance calculation setting). For the CC category, DEEPred was among the 10 best performing methods.

**Figure 2:**
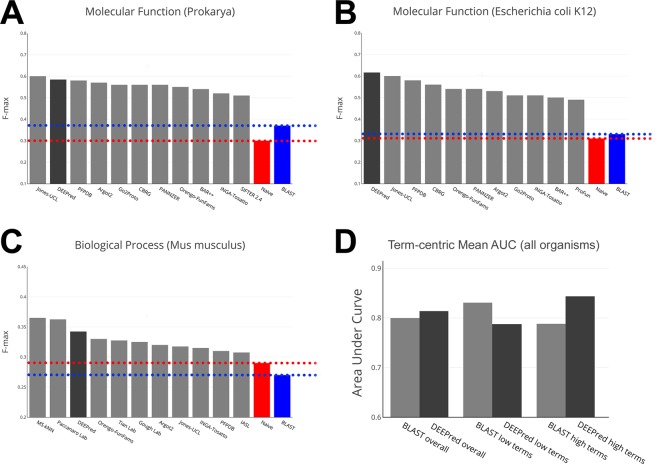
The prediction performance of DEEPred on CAFA2 challenge benchmark set. Dark gray colored bars represent the performance of DEEPred, whereas the light gray colored bars represent the state-of-the-art methods. The evaluation was carried out in the standard mode (i.e., no-knowledge benchmark sequences, the full evaluation mode), more details about the CAFA analysis can be found in CAFA GitHub repository; (**A**) MF term prediction performance (F-max) of top 10 CAFA participants and DEEPred on all prokaryotic benchmark sequences; (**B**) MF term prediction performance (F-max) of top 10 CAFA participants and DEEPred on E. coli benchmark sequences; (**C**) BP term prediction performance (F-max) of top 10 CAFA participants and DEEPred on mouse benchmark sequences; and (**D**) MF GO term-centric mean area under the ROC curve measurement comparison between BLAST and DEEPred for all MF GO terms, bars represent terms with less than 1000 training instances (i.e., low terms) and terms with more than 1000 training instances (i.e., high terms).

Figure 2A–C displays the 10 top performing methods in CAFA2 in terms of F-max measure along with the results of DEEPred, for the selected taxonomies where DEEPred performed well. As observed from Fig. 2A,B, DEEPred is among the best performers in terms of predicting MF GO terms for all prokaryotic sequences (Fig. 2A), specifically for *E. coli* (Fig. 2B). Figure 2C shows that DEEPred came third in predicting BP terms for the mouse (*Mus musculus*) proteins. These results (Fig. 2A–C) also indicate that DEEPred has an added value over the conventional baseline predictors (i.e., BLAST and naive). In Fig. 2D, we also compared our results with the BLAST baseline classifier in terms of the GO term-centric mean area under the ROC curve (AUC) for predicting MF terms for CAFA2 benchmark sequences. As it can be seen in Fig. 2D, the performance of DEEPred is slightly higher than the BLAST classifier in the overall comparison considering all MF GO terms. Whereas, the performance is low for DEEPred when MF GO terms of comparably low number of training instances (<1,000) was used (i.e., low terms). Finally, when the MF GO terms with comparably high number of training instances (>1,000) was employed (i.e., high terms), DEEPred’s performance surpassed BLAST. The results indicate that DEEPred is especially effective and have a significant added value over conventional methods, when the number of training instances are high.

The second performance analysis was done using CAFA3 challenge data, the submission period of which has ended in February 2017. The finalized benchmark dataset (protein sequences and their GO annotations) of CAFA3 was downloaded from the CAFA challenge repository on Synapse system (“benchmark20171115.tar” from https://www.synapse.org/#!Synapse:syn12278085). In total, this dataset contains 7,173 annotations (BP: 3,608, CC: 1,800 and MF: 1,765) for 3,312 proteins. The DEEPred models were re-trained using UniProt-GOA manual experimental evidence coded GO annotation data from September 2016 (the date of the official training data provided by CAFA3 organizers), and the predictions were generated for benchmark dataset protein sequences. For this analysis, we could not directly use the officially announced performance data of the top challenge performers since the results are yet to be published as of April 2019. Instead, three other sequence-based function prediction methods, namely FFPred3^36^, GoFDR^37^ and DeepGO^28^, were selected to be compared with DEEPred. These methods were developed and published in the last 2 years, and reported predictive performances that are better than the state-of-the-art in their own publications. For DeepGO, we downloaded the stand alone tool, train the models with the provided training data (considering the CAFA3 submission deadline) and produced the benchmark dataset predictions. The stand-alone tool was not available for FFPred3 and GoFDR; however, CAFA3 target set function predictions were already available, as a result, we directly employed those prediction files for our analysis. We also built baseline predictors (i.e., Naïve Bayes and Blast) with CAFA3 data, as described by the CAFA team. We employed performance evaluation scripts released by the CAFA team in order to calculate the performances of DEEPred, the state-of-the-art methods and the baseline classifiers. DEEPred is composed of multiple independent classifiers, each of which has its own best score threshold. For the calculation of F-max, CAFA evaluation script applies the same prediction score threshold to all predictions, which would result in the underestimation of DEEPred’s performance. To avoid this, we transformed DEEPred’s prediction scores and made them comparable to each other by applying min-max normalization.

Table 6 displays the performance results for Molecular function (MF), cellular component (CC) and biological process (BP) categories, in terms of F-max, precision, recall and Smin measures. Only the precision and recall values corresponding to the score threshold that produced the given F-max are shown. A better performance is indicated by higher F-max, precision and recall values and lower Smin values. “No-knowledge” and “All” indicates 2 different evaluation modes, where the former indicates that the methods are evaluated only using the proteins that did not have any manually curated GO annotation in the training dataset (before the challenge submission deadline), and the latter indicates that the methods are evaluated using all benchmark proteins. DEEPred was analzyed in terms of two different versions: *(i)* raw predictions coming from all predictive models, without any post-processing (i.e., DEEPred_raw), and *(ii)* finalized predictions after the hierarchical post-processing procedure (i.e., DEEPred_hrchy). The results are shown in Table 6, where the best results for each GO category and for each performance measure is highlighted with bold font. When the second best method’s performance was close to the best one, both of them are highlighted. As observed from Table 6, the finalized version of DEEPred (i.e., DEEPred_hrchy) consistently beat the performance of the raw DEEPred predictions, indicating the effectiveness of the proposed hierarchical post-processing approach. In MF term prediction, DEEPred_hrchy shared the top place with GoFDR in terms of F-max, precision and recall (GOFDR performed slightly better in terms of Smin). Considering CC term prediction, DeepGO shared the first place with the naïve classifier, in terms of both F-max and Smin. DEEPred_hrchy was the best (in terms of F-max) for predicting BP terms of the no-knowledge proteins, and shared the first place with DeepGO and FFPred3 considering all benchmark proteins. DEEPred_hrchy was also the first in terms of Smin, for the BP category. In CAFA3 analysis, at the points of maximum performance for DEEPred (i.e., Fmax) DEEPred’s precision was maximum (i.e., no FPs) and the recall was relatively low (i.e., high number of FNs), for all categories of GO, according to Table 6. The most probable reason behind this observation can be explained as follows. Since the multi-task modeling approach is used, the negative training instances/samples of a GO term in a model constitutes the positive training instances of other GO terms in the same model. In an example model where there are 5 GO terms, each with 50 positive training instances, there are 50 positive and 200 negative instances for each GO term. The ratio of 1 to 4 may lead to a bias towards negative predictions, especially for the cases where the proper learning was not achieved during training (i.e., a fully negative set of predictions would lead to success with a ratio of 4 to 5, whereas, a fully positive set of predictions would lead to successes with a ratio of 1 to 5). As a result, the system generally prefers fewer positive predictions, which usually means low number of FPs (high precision) and relatively higher number of FNs (low recall). Another probable reason behind this observation (for the finalized version of DEEPred) is that, the hierarchical post-processing of predictions reduces the number of false positives by eliminating predictions with low or moderate reliability (i.e., by checking the prediction consistency using the prediction results of the GO terms, which are the parents of the corresponding GO term). Thus, precision is increased due to discarding of some of the predictions; however, along with discarding FPs, some of the TPs were eliminated as well (i.e., those TPs turned into FNs), so the recall was reduced.

**Table 6:** The prediction performance of DEEPred and the state-of-the-art protein function prediction methods on CAFA3 challenge benchmark dataset.

	F-max		Precision		Recall (at F-max)		Smin	
	No-knowledge	All	No-knowledge	All	No-knowledge	All	No-knowledge	All
**MF**								
Naive	0.35	0.29	0.49	0.41	0.27	0.23	6.87	6.43
Blast	0.40	0.39	0.42	0.36	0.38	**0.44**	6.99	6.48
FFPred3	0.32	0.31	0.34	0.30	0.30	0.32	7.35	6.66
GoFDR*	**0.55**	0.45	0.67	0.55	**0.46**	0.38	**5.06**	**4.41**
DeepGO	0.40	0.34	0.58	0.48	0.30	0.27	6.36	6.01
DEEPred_raw	0.32	0.33	**1.00**	**1.00**	0.19	0.19	6.63	6.16
DEEPred_hrchy	0.49	**0.50**	**1.00**	**1.00**	0.32	0.33	5.41	5.03
**CC**								
Naive	**0.55**	**0.54**	0.56	0.58	0.55	0.50	**7.61**	**7.65**
Blast	0.46	0.45	0.39	0.39	0.56	0.53	9.74	9.94
FFPred3	0.54	0.52	0.54	0.54	0.53	0.50	8.61	8.44
GoFDR*	0.48	0.45	0.46	0.42	0.51	0.48	10.98	10.86
DeepGO	**0.54**	**0.53**	0.61	0.58	0.48	0.49	**7.68**	**7.55**
DEEPred_raw	0.30	0.29	0.19	0.18	**0.69**	**0.69**	10.68	10.41
DEEPred_hrchy	0.34	0.35	**1.00**	**1.00**	0.20	0.22	9.85	9.53
**BP**								
Naive	0.26	0.30	0.25	0.39	0.26	0.24	24.27	20.85
Blast	0.28	0.32	0.22	0.27	**0.37**	0.38	25.11	21.35
FFPred3	0.26	**0.34**	0.23	0.29	0.30	**0.40**	24.74	21.48
GoFDR*	0.19	0.18	0.25	0.26	0.15	0.14	24.75	28.83
DeepGO	0.28	**0.34**	0.40	0.52	0.21	0.26	23.41	20.19
DEEPred_raw	0.16	0.16	**1.00**	**1.00**	0.09	0.09	24.65	22.05
DEEPred_hrchy	**0.32**	**0.33**	**1.00**	1.00	0.19	0.19	**22.04**	**19.69**

*The results of GoFDR is given based on the CAFA3 preliminary benchmark set since the results for the full benchmark dataset were not available for this method.

### *P. aureginosa* Case Study on biofilm formation process

We analyzed the biological relevance of the results of DEEPred over selected example predictions. For this purpose, we employed the recent CAFA Pi biological process GO term assignment challenge. One of the goals in CAFA Pi was the prediction of the proteins responsible for the biofilm formation (GO:0042710) process using electronically translated open reading frames (ORFs) from a specific *Pseudomonas aureginosa* strain (UCBPP-PA14) genome. A short introduction about *P. aureginosa* and biofilm formation process can be found in the Supplementary Material 1 Document, Section 8.

In order to annotate ORF sequences from *P. aureginosa* UCBPP-PA14 strain with biofilm formation GO term using DEEPred, we generated a single task feed-forward DNN model. The reason behind not using a multi-task model here was to prevent the potential effect of the selection of the accompanying GO terms to the predictive performance. The positive training dataset for this model was generated from all UniProtKB/Swiss-Prot protein records that were annotated either with the corresponding GO term or with its descendants with manual and experimental evidence codes, yielding 254 proteins. The negative training dataset was selected from the protein entries that were neither annotated with the corresponding GO term nor any of its descendants (the same number of samples were selected randomly to match the positive training dataset). The model was trained, and the hyper-parameters were optimized and the performance was measured via 5-fold cross validation. The performance results in terms of precision, recall and F1-score were 0.71, 0.84 and 0.77 respectively. The finalized models were then employed to predict functions for CAFA Pi *P. aureginosa* ORF targets.

From a literature review, we identified 8 genes (wspA, wspR, rocR, yfiN, tpbB, fleQ, fimX and PA2572) in the *P. aureginosa* reference genome that are associated with biofilm formation, but not annotated with the corresponding GO term or its functionally related neighboring terms, in the source databases at the time of this analysis (as a result, they are not presented in our training dataset). Out of these 8 genes/proteins wspR, yfiN, tpbB and fimX contain the GGDEF domain, which is responsible for synthesizing cyclic di-GMP and thus take part in the biofilm formation process^38^. Two of these genes/proteins, yfiN and tpbB, additionally contain the CHASE8 sensor domain, which controls the levels of extracellular DNA and regulates biofilm formation^39^. The mechanism by which these 8 genes/proteins contribute to the formation of biofilm are explained in two articles by Cheng^40^ and Ryan *et al*.^41^. We obtained the protein sequences of these genes from the UniProt database, then aligned them to CAFA Pi *P. aureginosa* UCBPP-PA14 strain’s target ORF sequences to identify CAFA Pi target sequences corresponding to these genes with a cut-off of greater than 98% identity. The reason behind this application was that the CAFA Pi target dataset ORF sequences were unknown. Finally, we analyzed the equivalent *P. aureginosa* ORFs of these 8 genes in the target dataset using DEEPred’s biofilm formation process model and examined the prediction scores.

Table 7 displays the gene symbols, protein (UniProt) accessions and biofilm formation GO term prediction scores produced by DEEPred for the selected genes/proteins. As observed in Table 7, 4 out of 8 genes/proteins (i.e., gene symbols: wspA, wspR, rocR and PA2572) received high prediction scores for the biofilm production term and thus successfully identified by DEEPred. Two genes/proteins (i.e., gene symbols: yfiN and tpbB) received moderate scores, which were still sufficient to produce a prediction. The remaining two genes/proteins (i.e., gene symbols: fleQ and fimX) could not be associated with the corresponding GO term at all. We also carried out a BLAST search in order to observe if these predictions could be produced by a conventional sequence similarity search. For this, the amino acid sequence of each of the 8 genes/proteins was searched against the whole UniProtKB with an e-value threshold of 100. The BLAST search revealed that none of the best 1,000 BLAST hits (50% or greater identity) possessed the biofilm formation GO term or any of its ancestor or descendant terms as annotations, and thus BLAST failed to annotate these genes/proteins. Since none of these 8 genes/proteins (or their BLAST hits) have been annotated with a GO term related to the biofilm formation function on the GO DAG; there were no protein sequences in the training dataset of DEEPred that were similar to these genes. As a result, the accurate predictions cannot be the result of a simple annotation transfer between close homologs.

**Table 7:** DEEPred’s biofilm formation term (GO:0042710) prediction results for the selected *P. aureginosa* proteins.

Gene symbol	Protein accession (UniProt)	DEEPred prediction score
wspA	A0A0H2ZEY3	0.99
rocR	A0A0C7D525	0.98
PA2572	Q9I0R4	0.98
wspR	A0A0H2ZEX4	0.95
yfiN	A0A0C7ADU5	0.68
tpbB	Q9I4L5	0.68
fleQ	A0A0H2Z7X4	0.05
fimX	A0A0H2ZHA6	0.02

## Discussion and Conclusion

Deep learning algorithms have shown to enhance the classification performances in various fields; however, it was not thoroughly investigated in terms of their applications to the protein function prediction area at large-scale. In this study, we described the DEEPred method for predicting GO term based protein functions using a stack of feed-forward multi-task deep neural networks. As input, DEEPred only requires the amino acid sequences of proteins. We carried out several tests to investigate the behavior of DNN-based models in protein function prediction. The approach developed and applied in this study is novel in terms of:

i. timeliness of the work: the application of deep learning based methods on different bioinformatics related problems is currently a hot topic, the pre-print of DEEPred was one of the first in the protein function prediction literature^35^;
ii. technical contribution in terms of designing a novel DNN-based system: 1,101 multi-task feed-forward deep neural networks are constructed, individually optimized, and stacked according to the inheritance relationships of the Gene Ontology system, which enables a hierarchical prediction and post-processing process;
iii. a thorough investigation of the behavior of deep neural networks when applied to GO-based protein function prediction, considering different input feature types, dataset sizes and GO term levels;
iv. data modelling approach: electronically made annotations (i.e., predictions of previous protein function prediction methods) are included in the training set of the predictor, with the aim of enriching training data (especially for the GO terms with insufficient number of training instances), and the results are examined in detail; as far as we are aware, this investigation is the first in protein function prediction literature;
v. DEEPred contributes to the state of the art in the field of protein function prediction, in terms of designing a large-scale deep learning based system that is able to model thousands of GO terms, which is also practical to use.

The input feature type selection analysis revealed that our in-house protein descriptor SPMap had a better performance compared to the conventional conjoint triad and pseudo-amino acid composition features. However, this performance increase comes with a cost in terms of a higher vector dimensionality (i.e., SPMap has between 1000 to 2000 dimensions as opposed to 373 for conjoint triad and 50 for pseudo-amino acid composition), which elevates the computational complexity. It would also be interesting to analyze additional protein feature types, especially the descriptors frequently used in protein-ligand binding prediction studies^42^.

The reasons behind choosing DEEPred’s specific DNN architecture was first, this is a basic form and thus, it is straightforward to train and optimize. In other words, it requires minimal amount manual design work compared to specialized complex networks such as the Inception Network^43^. This is especially important considering the fact that more than one thousand independent networks should have been trained. Second, computational resources required to train this architecture is lower compared to, again, complex networks.

In DEEPred, we considered multi-task DNNs (as opposed to single-task DNNs) due to various advantages attributed to multi-task networks such as: *(i)* the ability to share knowledge between tasks; which supports the system in the case where there are a low number of training instances and *(ii)* training a lower number of models in total, which improves training run times. On the other hand, multi-task DNNs also have disadvantages especially when the high number of tasks compels the generation of multiple models. The problem here is the efficient grouping of the tasks (i.e., GO terms in our case) so that the tasks under an individual model would become alternatives (i.e. orthogonal) to each other. We tried to achieve this by first, grouping GO terms from the same level and second, placing the sibling terms together under the same model, where possible. In most cases, it was not possible to find a sufficient number of sibling terms, and thus, semantically unrelated terms from the same level ended up in the same model. Nevertheless, this was not a crucial problem since it is possible for a multi-functional query protein to receive high prediction scores for multiple GO terms under the same model. Another important point during the term grouping was placing GO terms with similar number of annotated proteins under the same group. According to our observations, models containing tasks with highly unbalanced number of training instances perform poorly (this is also one of the reasons why generating only one model to predict all GO terms would be a poor design choice). Due to these reasons, generation of the models required a considerable amount of manual work, none of which would be required if we employed single-task networks. It would also be possible to achieve higher performance values with single-task DNNs, especially where there is sufficient number of training instances. We did not consider single-task networks mainly because it is not feasible to train tens of thousands of networks (when the hyper-parameter optimization step is considered the number would increase to billions of training jobs) to cover the whole functional space. In the future, it would be interesting to see algorithmic solutions to the feasibility problems related to single-task networks. With such solutions we could construct and test a single-task DNN-based system for protein function prediction.

In this study, we trained several DNN models using 6 different groups of training datasets containing GO terms with differing number of training samples, to investigate the performance differences due to changes in the training sample size. Our training dataset size performance evaluation results (Fig. 1 and Table 3) showed that there is a general trend of performance increase with the increasing number of training samples, which means that including GO terms with low number of protein associations into models decreases the overall performance. Therefore, our findings are in accordance with the literature regarding training data sizes being one of the key factors that affect the predictive performance of deep learning algorithms; though, the research community started to focus on developing novel deep learning based approaches to address training dataset size related problems^44^.

In this work, we also investigated if there is a relationship between levels of GO terms on the GO DAG and the classification performances. Figure S.1 indicates that there is no such correlation. In addition, we observed that the variance in performance between different GO levels decreases as the training dataset size increases for molecular function and cellular component categories. For the biological process category, the overall performance increases with increasing GO training dataset sizes, however the variance is relatively higher. The main reason behind this may be attributed to the biological process GO terms representing complex processes (e.g., GO:0006099 - tricarboxylic acid cycle) that involves several molecular events, which is hard to associate with a sequence signature. Figure S.1 also showed that performance variance of cellular component GO terms is lower compared to the molecular function and biological process categories. The reason for such observation could be that the hierarchy between cellular compartment GO terms is inherently available within cells, which results in well defined hierarchical relationships between cellular component GO terms.

In most of the protein function prediction methods, training was performed using only the annotations with experimental and/or manual evidence codes. The disadvantage of this approach is that most GO terms are left with a small number of annotated proteins, which is usually not sufficient for a machine learning model training. Therefore, the functions defined by these terms cannot be predicted efficiently. One solution would be to include the annotations with no-curation evidence codes such as the electronic annotations (i.e., the annotations produced by other automated approaches). For example, the number of MF GO terms that have more than 30 protein associations is calculated as 911 when we only considered the annotations with manual and experimental evidence codes. However, when we considered the annotations with all evidence codes, this number increases to 2,776, meaning that, if the annotations with all evidences are included, it is possible to provide predictions for significantly more GO terms. The main downside of adding annotations with non-manual/experimental evidence codes to the training dataset is the potentially false positive samples, which would result in error propagation. Another potential limitation of this application would be that the predictive performance of the models with training datasets dominated by the electronic annotations would still be low (even though the number of training instances are increased), due to the fact that the sequences of most of the electronically annotated genes/proteins under a distinct GO term would be extremely similar to each other (due to annotation by sequence similarity); and thus, would not provide the required sample diversity.

At the end of the training dataset enrichment analysis, the evaluation results showed that the performance of the previously low performing GO term models were increased significantly (Table 4), which indicates that deep learning algorithms are tolerant to noise in the learning data. Therefore, annotations with less reliable evidence codes can be included in the training of low performing models, where there is still room for significant performance improvement. However, including less reliable annotations in the training dataset of previously high performaning models decreased the performance for more than half of them.

In DEEPred, we employed a hierarchical post-processing method (in order to avoid false positive hits) by taking the prediction scores of the parents of the target GO term into account, along with the actual prediction score for the target term. The evaluation results indicated that the recall values were slightly decreased and the precision scores were noticeably increased when we employed the hierarchical post-processing procedure, producing an increased overall performance in terms of F1-score (Table 5). In this setting, the resulting predictions can be considered more reliable. This is also indicated by the improved F-max values at the CAFA3 benchmark test (Table 6).

In our performance tests, DEEPred performed slightly better than the state-of-the-art methods in some cases, and produced roughly similar results in others. However, we did not observe an unprecedented performance increase; probably because we did not focus on specific functional families to optimize the system performance. Instead, we investigated the applicability of DNNs for constructing large-scale automated protein function prediction pipelines. We believe that this investigation will be valuable for computational scientists in terms of developing DNN-based biological data prediction methods. According to our observations, it is feasible to use DNNs in large-scale biological data analysis pipelines, where it may be possible to achieve performances higher than the state-of-the-art, with further optimization. However, feed-forward DNN based modeling is probably not a good choice for the functional terms with low or moderate number of annotated proteins (at least without a pre-processing step such as the training dataset enrichment), for which, conventional machine learning solutions or DNN methods specialized in low-data training may be considered.

Generally, function prediction methods that incorporate multiple types of protein features at once (e.g., sequence, protein-protein interactions - PPIs, 3-D structures and annotations and etc.) perform better compared to methods that incorporate sequences, solely^9,10^. However, there are two main disadvantages of this approach. First of all, query proteins are required to have a substantial amount of characterization (especially in terms of PPIs and 3-D structures) in order for these methods to accept them as queries. Structurally well characterized proteins usually have high quality functional annotations, thus, function prediction methods are not required in the first place. Second, running times of these methods are generally multiple orders of magnitude higher compared to the sequence-based predictors, which significantly hinders their large-scale use, such as the analysis of newly sequenced genomes.

Finally in this study, we carried out a case study to discuss the biological relevance of the results produced by DEEPred, by predicting the *Pseudomonas aureginosa* ORF sequences that take part in the biofilm formation biological process. DEEPred managed to identify 6 out of 8 proteins that are reported to play roles in the biofilm formation function, which are not annotated with the corresponding GO term (or any of its descendent terms) in the source biological databases as of April 2018. As a result, it can be said that without any prior knowledge DEEPred produced biologically relevant predictions considering the selected function. It is also evident that DEEPred performed significantly better in this test, compared to the baseline classifier (i.e., BLAST). It is difficult to identify how deep neural networks managed to annotate these proteins where BLAST failed significantly; however, it can be attributed to DNN’s ability to extract signatures (relevant to the task at hand) hidden in the sequences, by consequent levels of data abstraction.

The methodological approach proposed in this study can easily be translated into the prediction of various types of biomolecular ontologies/attributes (e.g., protein families, interactions, pathways, subcellular locations, catalytic activities, EC numbers and structural features) and biomedical entity associations (e.g., gene-phenotype-disease relations and drug-target interactions).

## Materials and Methods

### Training dataset construction

The training dataset of DEEPred was created using the UniProtKB/Swiss-Prot database (version 2017_08) protein entries. UniProt supports each functional annotation with one of the 21 different evidence codes, which indicate the source of the particular annotation. In this study, we used annotations with manual curation or experimental evidences, which are considered to be highly reliable. In order to generate the training dataset, the corresponding annotations were extracted from the UniProt-GOA database, propagated to their parent terms according to the “*true path rule*”, which defines the inheritance relationship between GO terms^2^. Using this dataset, a positive training dataset was constructed for each GO term. In short, proteins that are annotated either with the corresponding GO term or with one of its children terms, were included in the positive training dataset of the corresponding GO term. Since our multi-task DNN models are composed of multiple GO terms, the positive training instances for one GO term, in a model, constitute the negative training instances of the other GO terms in the same model, except the proteins that are annotated with both GO terms.

In order to analyze the effect of the extent of training datasets on the predictive performance, we constructed multiple “training-set-size-based” datasets, taking into account the number of protein associations of GO terms. For example, one of our training-set-size-based datasets includes all GO terms that have more than or equal to 30 protein associations. Hence, we created six different datasets, where GO terms in each dataset have greater than 30, 100, 200, 300, 400 and finally 500 protein associations, respectively. These datasets comprise each other (e.g., GO-terms-with-greater-than-30-proteins dataset covers the GO-terms-with-greater-than-100-proteins dataset). The statistics (i.e., number of annotations, GO terms and proteins) of these datasets are given in the Results section.

### DEEPred architecture

**Figure 3:**
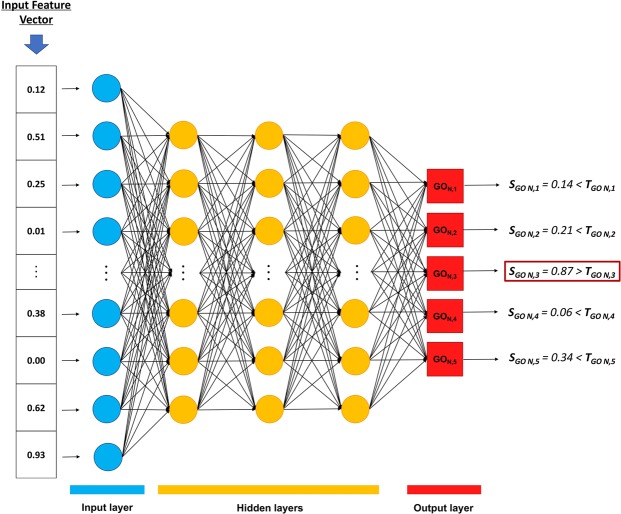
The representation of an individual multi-task feed-forward DNN model of DEEPred (i.e., model N). Here, each task at the output layer (i.e., red squares) corresponds to a different GO term. In the example above, a query input vector is fed to the trained model N and a score greater than the pre-defined threshold is produced for GON,3, which is marked as a prediction.

DEEPred was built as a stack of multi-task feed-forward deep neural networks (i.e., a stack of multi-task multi-layered perceptrons), connected to each other. In DEEPred, each DNN was independently modelled to predict 4 or 5 GO terms, thus multiple DNNs were required to cover thousands of terms. Figure 3 displays a representative DNN model in DEEPred.

**Figure 4:**
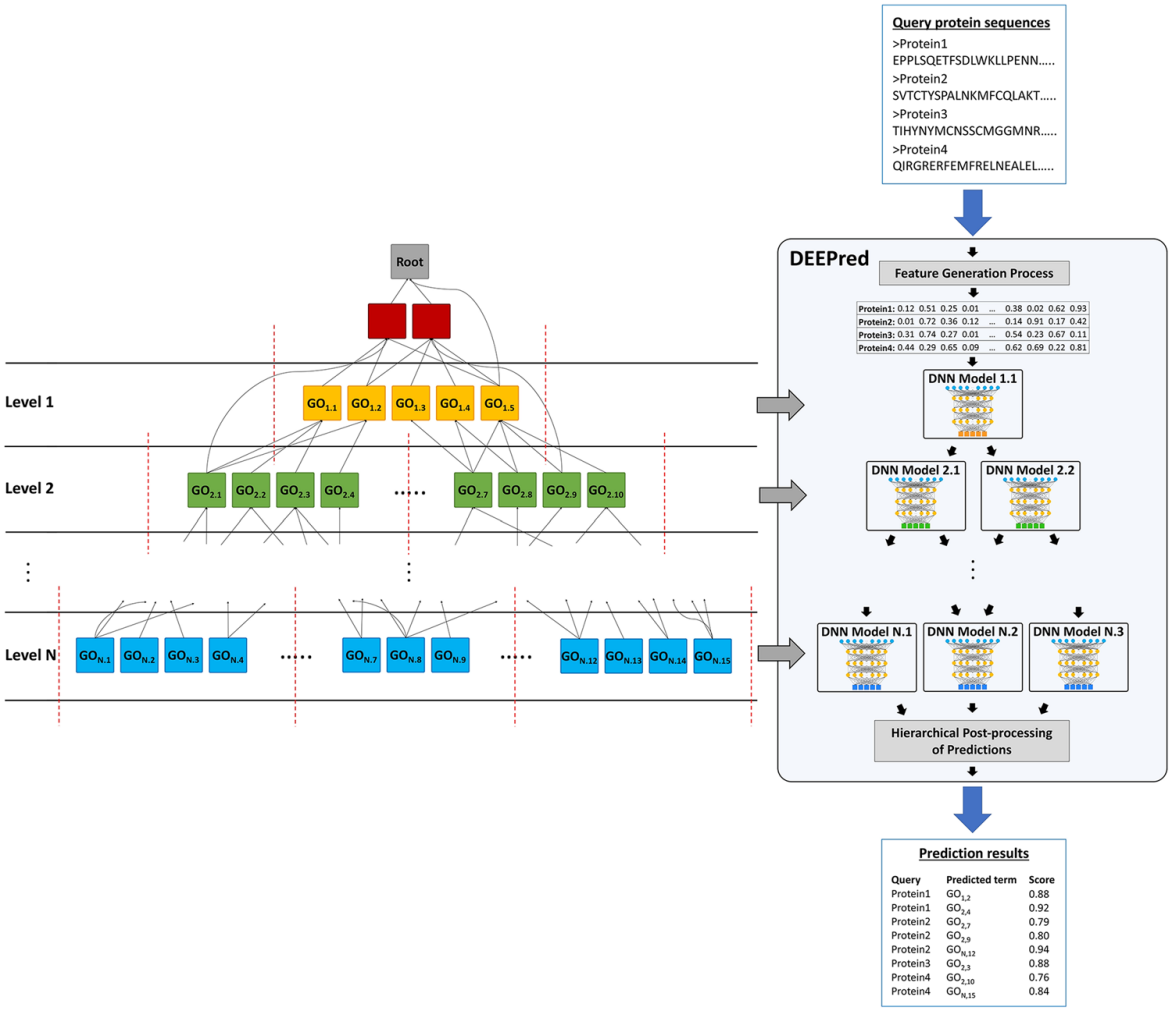
Illustration of the GO-level-based architecture of DEEPred on a simplified hypothetical GO DAG. We omitted highly generic GO terms (shown with red colored boxes) at the top of the GO hierarchy (e.g., GO:0005488 - Binding) from our models, since they are less informative and their training datasets are highly heterogeneous. In the illustration, DNN model 1.1 incorporates GO terms: GO_1,1_ to GO_1,5_ from GO-level 1. In the real application, most of the GO levels were too crowded to be modeled in one DNN; in these cases, multiple DNN models were created for the same GO level (red dashed lines represent how GO terms are grouped to be modeled together). In this example, DNN models N.1, N.2 and N.3 incorporates GO terms: GO_N,1_ to GO_N,5_, GO_N,6_ to GO_N,10_, GO_N,11_ to GO_N,15_; respectively, due to the high number of GO terms on level N. At the prediction step, when a list of query sequences is run on DEEPred, all sequences are transformed into feature vectors and fed to the multi-task DNN models. Afterwards, GO term predictions from each model are evaluated together in the hierarchical post-processing procedure to present the finalized prediction list.

The selection of GO terms for each DNN model was based on the levels of the terms on the GO DAG. The main objective of this approach was to create a multi-task deep neural network model for each level. For this, the levels of all GO terms were extracted and the terms were separated into groups based on the level information (via topological sorting). We started the level numbering from generic terms; thus, they received low numbers (e.g., level 1, 2, 3, …) and the levels of specific terms received high numbers (e.g., level 10, 11, 12, …). In most cases, the number of protein associations of GO terms within a level were highly variable; therefore, we created subgroups to further avoid bias (i.e., tendency of a classifier to give predictions to classes with significantly higher number of training instances). Here, each subgroup included GO terms with similar number of annotations. Another reason behind generating multiple models under a specific GO level was the high number of GO terms. According to our tests, when the number of tasks under a model exceed 5 or 6, the models usually perform poorly. Due to this reason, we limited the number of tasks under a model to 5 in most cases. This procedure generated 1,101 different models concerning all GO categories. Figure 4 represents the GO-level-based arrangement of the individual DNN models in DEEPred. Supplementary Material 2 lists the GO terms at each GO level, and at each sub-level model (when a GO level is further divided to multiple models).

In a feed-forward DNN, forward propagation (*z*) for the layer *l* is calculated by the following equation:

1 $${z}^{l}={b}^{l}+{W}^{l}\,\ast \,{a}^{l-1}$$

where *b*^*l*^ is the bias vector, *W*^*l*^ is the weight matrix for the *l*^*th*^ layer and *a*^*l*−1^ is the activation value vector of the neurons at the previous layer. Subsequently, an activation function, *g*^*l*^(*), is applied to the calculated *z*^*l*^ vector and the result of the activation function is used to compute the outputs of the *l*^*th*^ layer:

2 $${a}^{l}={g}^{l}({z}^{l})$$

3 $${g}^{l}=max(0,{z}^{l}\,)$$

There are alternative activation functions such as sigmoid, *tanh* and rectified linear unit (ReLU). Here, we employed ReLU activation function for the hidden layers. The prediction scores are calculated by applying *softmax* function to the neurons at the output layer. The score for the *j*^*th*^ task is calculated by the following equation:

4 $${S}_{j}=\frac{{e}^{j}}{{\sum }_{k}{e}^{k}}$$

where *k* is the number of tasks to be trained within a model. At the end of the forward propagation step, prediction scores are used to calculate a cost function, *C*, based on the labels of the input samples. In this study, we used cross entropy to calculate the cost function. Once the cost function is calculated, it is used to determine how much the weights (*w*) will be changed after the last iteration by taking the partial derivatives of the cost function with respect to the weights:

5 $${w}_{i}={w}_{i}-\eta \frac{\partial C}{\partial {w}_{i}}$$

where *η* is the learning rate in the equation. The forward and back propagation steps were performed until the stopping criteria was met (e.g., after certain number iterations or after the objective performance is reached). For the training of the models of DEEPred, 100 iterations were selected.

In DEEPred, each model was independently trained using the feature vectors of the proteins annotated with the corresponding GO terms of that model. Considering the technical work to accomplish the multi-task training, we created a binary “true label vector” for each protein sequence using one-hot encoding, where each dimension represented a GO term to be trained in the corresponding model. The index of the GO term that was associated with the corresponding protein sequence was set to 1 and the remaining dimensions were set to 0. These true label vectors were employed to calculate the prediction errors at the output layer, which was then used by the optimizer to update weights with the aim of minimizing prediction error at each iteration. The hyper-parameters of the predictive models in DEEPred are explained in the Supplementary Material 1 Document, Section 2. The results of the hyper-parameter optimization test are explained in Supplementary Material 1, Section 6; whereas, the actual comprehensive test results are given in Supplementary Material 3.

At the prediction stage, a query protein sequence feature vector is first fed to the level 1 predictor model to receive its probabilistic scores for the corresponding GO terms and then fed to the level 2 predictor model to receive probabilistic scores for a different set of GO terms. At the end of the process, GO terms that obtained scores above the pre-determined thresholds were fed to the hierarchical post-processing (explained below under the section entitled: “Hierarchical Post-processing of Predictions”) and the finalized predictions were produced (Fig. 4).

### Protein feature types and vector generation

In order to select the best protein feature representation for DEEPred, we implemented three alternative protein descriptor generation methods: *(i)* Conjoint triad^45^, *(ii)* Pseudo amino acid composition^46^ and *(iii)* Subsequence profile map (SPMap)^47^. Each of these feature types were used individually to train and to test the system. These protein feature types are explained in the Supplementary Material 1 Document, Section 4. The details about this analysis is given in the Results section.

### Determining the probabilistic score thresholds

When a query protein is fed to a prediction model of DEEPred, an individual probabilistic score is calculated for each GO term (i.e., task) within that model, representing the probability of the query protein possessing the function defined by the corresponding GO term (Fig. 3). In some cases, this can be confusing because scores are on a continuous scale (i.e., it is not clear at which point one can conclude that the query protein contains the corresponding function). Usually, the requirement from a model is to make a binary prediction instead of producing a score. Setting a probabilistic score threshold for each GO term at each model solves this problem. At the prediction step, if the received score is equal to or greater than the pre-defined threshold, the model outputs a positive prediction for the corresponding GO term. To determine these thresholds in a validation setting (using the hold-out validation datasets), we calculated F1-score performance values for arbitrary threshold selections using the success of the binary predictions obtained when we fed the system with protein sequences with already known labels (i.e., GO term associations). We considered each GO term separately within a model and determined an individual threshold for each term by choosing the value providing the highest F1-score. These threshold values are stored in ready-to-use predictive models of DEEPred.

### Hierarchical post-processing of predictions

We implemented a methodology to eliminate the unreliable predictions by considering the prediction scores received for the parents of the target GO term. This way, we aimed to reduce the amount of false positive hits. The reason behind the requirement for such a post-processing step was that, multi-task DNNs tended to classify query instances to at least one of the tasks at the output layer. Such a classification scheme would not be a problem if we could generate one model that contain all of the GO terms at its output layer. However, having thousands of nodes in the output layer would be highly impractical and thus we divided GO terms into different models. This time, the problem occurs when a query protein is fed to a model, where the protein does not contain any of the functions defined by the GO terms in the corresponding model. The model often predicts one of the unrelated GO terms for the query protein, producing a false positive. We observed that separating a false positive hit (produced this way) from a reliable prediction would be possible by checking the prediction results for the parents of the predicted GO term. If the query protein consistently received high prediction scores for most of the parent terms as well, we can conclude that this case is probably a reliable prediction; otherwise, it may be a false positive hit.

**Figure 5:**
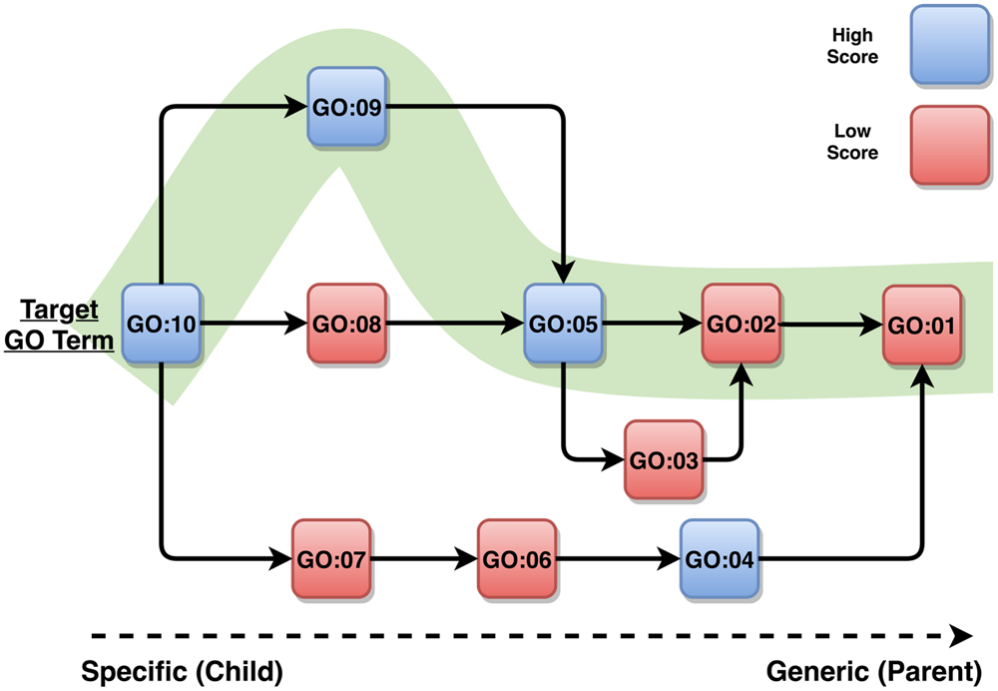
Post-processing of a prediction (GO:10) for a query protein sequence on a hypothetical GO DAG. Each box corresponds to a different GO term, with identification numbers written inside. The blue colored boxes represent GO terms whose prediction scores are over the pre-calculated threshold values (i.e., predicted terms), whereas the red colored boxes represent GO terms, whose prediction scores are below the pre-calculated threshold values (i.e., non-predicted terms). The arrows indicate the term relationships. There are four different paths from the target term (i.e., GO:10) to the root (i.e., GO:01) in this hypothetical DAG. Since there is at least one path, where the majority of the terms received higher-than-threshold scores (shown by the shaded green line), the target term GO:10 is given as a finalized positive prediction for the query sequence.

To construct this methodology, we first topologically sorted the DAG for each GO category and determined all possible paths from each GO term to the root of the corresponding category, and stored this information. When a query protein is run on DEEPred, its feature vector is fed to all trained models to obtain the prediction scores for all GO terms. Starting from the most specific level of GO, the method checks whether the prediction score of the query protein is greater than the previously calculated score thresholds. If the prediction score of a target GO term is greater than its threshold, the method checks the scores it received for the parent terms on all paths to the root, using the previously stored possible-paths-to-root. If the prediction scores given to the majority of parent terms are greater than their individual thresholds (in at least one of the paths), the method presents the case as a positive prediction. This procedure is represented in Fig. 5 with a toy example.

### Predictive performance evaluation tests

According to the current deep learning practice, it is not feasible to carry out a fold-based cross validation analysis, especially when the number of model training operations are high, since it usually requires extremely high computational power. This issue was also valid for DEEPred due to the presence of elevated number of models. For this reason, the assessment of DEEPred system was performed using three datasets: *(i)* a hold-out validation dataset, *(ii)* CAFA2, and *(iii)* CAFA3 challenge benchmark datasets. The preparation of these datasets are explained in the Supplementary Material 1 Document, Section 5. Performance evaluation metrics used in this study are explained in the Supplementary Material 1 Document, Section 3.

## Supplementary information

Supplementary_Material_1 (Document 1)

Supplementary_Material_2 (Dataset 1)

Supplementary_Material_3 (Dataset 2)

## Data Availability

The source code and all datasets used in this study are available at: https://github.com/cansyl/DEEPred.

## References

[1] Consortium TU (2016). UniProt: the universal protein knowledgebase. Nucleic Acids Res..

[2] Blake JA (2015). Gene ontology consortium: Going forward. Nucleic Acids Res..

[3] Rifaioglu AS (2017). Large-scale automated function prediction of protein sequences and an experimental case study validation on PTEN transcript variants. Proteins Struct. Funct. Bioinforma..

[4] Doğan T (2016). UniProt-DAAC: domain architecture alignment and classification, a new method for automatic functional annotation in UniProtKB. Bioinformatics.

[5] Lan L, Djuric N, Guo Y, Vucetic S (2013). MS-kNN: protein function prediction by integrating multiple data sources. BMC Bioinformatics.

[6] Wass MN, Barton G, Sternberg MJE (2012). CombFunc: Predicting protein function using heterogeneous data sources. Nucleic Acids Res..

[7] Tiwari AK, Srivastava R (2014). A survey of computational intelligence techniques in protein function prediction. Int. J. Proteomics.

[8] Koskinen P, Törönen P, Nokso-Koivisto J, Holm L (2015). PANNZER: High-throughput functional annotation of uncharacterized proteins in an error-prone environment. Bioinformatics.

[9] Jiang Y (2016). An expanded evaluation of protein function prediction methods shows an improvement in accuracy. Genome Biol..

[10] Radivojac P (2013). A large-scale evaluation of computational protein function prediction. Nat. Methods.

[11] Anderson, J. A. *An introduction to neural networks*. (MIT Press, 1995).

[12] Hinton Geoffrey, Deng Li, Yu Dong, Dahl George, Mohamed Abdel-rahman, Jaitly Navdeep, Senior Andrew, Vanhoucke Vincent, Nguyen Patrick, Sainath Tara, Kingsbury Brian (2012). Deep Neural Networks for Acoustic Modeling in Speech Recognition: The Shared Views of Four Research Groups. IEEE Signal Processing Magazine.

[13] Deng, L., Hinton, G. & Kingsbury, B. New Types of Deep Neural Network Learning For Speech Recognition And Related Applications: An Overview 1–5 (2013).

[14] Angermueller C (2016). Deep Learning for Computational Biology. Mol. Syst. Biol..

[15] Min S, Lee B, Yoon S (2016). Deep learning in bioinformatics. Brief. Bioinform..

[16] Taigman, Y., Ranzato, M. A., Aviv, T. & Park, M. Deepface 1–8, 10.1109/CVPR.2014.220 (2014)

[17] Lecun Y, Bengio Y, Hinton G (2015). Deep learning. Nature.

[18] Gawehn E, Hiss JA, Schneider G (2016). Deep Learning in Drug Discovery. Mol. Inform..

[19] Baskin II, Winkler D, Tetko IV (2016). A renaissance of neural networks in drug discovery. Expert Opin. Drug Discov. ISSN.

[20] Mayr A, Klambauer G, Unterthiner T, Hochreiter S (2016). DeepTox: Toxicity Prediction using Deep Learning. Front. Environ. Sci..

[21] Ramsundar, B. *et al*. Massively Multitask Networks for Drug Discovery arXiv:1502.02072v1. *arXiv* 1–27 (2015).

[22] Bengio, Y. *Learning Deep Architectures for AI*. *Foundations and Trends® in Machine Learning* **2** (2009).

[23] Goh GB, Hodas NO, Vishnu A (2017). Deep Learning for Computational Chemistry. arXiv.

[24] Saberi Mohamad Mohd, Rocha Miguel P., Fdez-Riverola Florentino, Domínguez Mayo Francisco J., De Paz Juan F. (2016). 10th International Conference on Practical Applications of Computational Biology & Bioinformatics.

[25] Sliwoski G, Kothiwale S, Meiler J, Lowe EW (2014). Computational Methods in Drug Discovery. Pharmacol. Rev..

[26] Liu, X. L. Deep Recurrent Neural Network for Protein Function Prediction from Sequence. *arXiv* 1–38 (2017).

[27] Cao Renzhi, Freitas Colton, Chan Leong, Sun Miao, Jiang Haiqing, Chen Zhangxin (2017). ProLanGO: Protein Function Prediction Using Neural Machine Translation Based on a Recurrent Neural Network. Molecules.

[28] Kulmanov M, Khan MA, Hoehndorf R (2017). DeepGO: Predicting protein functions from sequence and interactions using a deep ontology-aware classifier. Bioinformatics.

[29] Szalkai B, Grolmusz V, Hancock J (2018). SECLAF: A Webserver and Deep Neural Network Design Tool for Hierarchical Biological Sequence Classification. Bioinformatics.

[30] Tavanaei, A. *et al*. Towards Recognition of Protein Function based on its Structure using Deep Convolutional Networks. *IEEE Int. Conf. Bioinforma. Biomed*. 145–149, 10.1109/BIBM.2016.7822509 (2016).

[31] Gligorijević, V., Barot, M. & Bonneau, R. DeepNF: Deep network fusion for protein function prediction. *bioRxiv* **223339**, 10.1101/223339 (2017). 10.1093/bioinformatics/bty440 PMC6223364 29868758

[32] Fa, R., Cozzetto, D., Wan, C. & Jones, D. T. Predicting Human Protein Function with Multi-task Deep Neural Networks. *bioRxiv* (2018). 10.1371/journal.pone.0198216 PMC5995439 29889900

[33] Chicco D, Sadowski P, Baldi P (2014). Deep autoencoder neural networks for gene ontology annotation predictions. Proc. 5th ACM Conf. Bioinformatics, Comput. Biol. Heal. Informatics - BCB.

[34] Zou X, Wang G, Guoxian Y (2017). Protein Function Prediction Using Deep Restricted Boltzmann Machines. BioMed Res. Int..

[35] Rifaioglu, A. S., Doğan, T., Martin, M. J., Cetin-Atalay, R. & Atalay, M. V. Multi-task Deep Neural Networks in Automated Protein Function Prediction. *arXiv* 1–19 (2017). 10.1038/s41598-019-43708-3 PMC6517386 31089211

[36] Cozzetto D, Minneci F, Currant H, Jones DT (2016). FFPred 3: Feature-based function prediction for all Gene Ontology domains. Sci. Rep..

[37] Gong Q, Ning W, Tian W (2016). GoFDR: A sequence alignment based method for predicting protein functions. Methods.

[38] Ryjenkov DA, Tarutina M, Moskvin OV, Gomelsky M (2005). Cyclic diguanylate is a ubiquitous signaling molecule in bacteria: Insights into biochemistry of the GGDEF protein domain. J. Bacteriol..

[39] Ueda A, Wood TK (2009). Connecting quorum sensing, c-di-GMP, pel polysaccharide, and biofilm formation in Pseudomonas aeruginosa through tyrosine phosphatase TpbA (PA3885). PLoS Pathog..

[40] Chang C-Y (2018). Surface Sensing for Biofilm Formation in Pseudomonas aeruginosa. Front. Microbiol..

[41] Ryan RP (2009). HD-GYP domain proteins regulate biofilm formation and virulence in Pseudomonas aeruginosa. Environ. Microbiol..

[42] Van Westen GJP (2013). Benchmarking of protein descriptor sets in proteochemometric modeling (part 1): comparative study of 13 amino acid descriptor sets. J. Cheminform..

[43] Szegedy C (2015). Going deeper with convolutions. Proc. IEEE Comput. Soc. Conf. Comput. Vis. Pattern Recognit..

[44] Altae-Tran H, Ramsundar B, Pappu AS, Pande V (2017). Low Data Drug Discovery with One-Shot Learning. ACS Cent. Sci..

[45] Shen J (2007). Predicting protein-protein interactions based only on sequences information. Proc. Natl. Acad. Sci. USA.

[46] Chou K-C (2001). Prediction of Protein Cellular Attributes Using Pseudo- Amino Acid Composition. Proteins Struct., Funct., Genet..

[47] Sarac OS, Gürsoy-Yüzügüllü O, Cetin-Atalay R, Atalay V (2008). Subsequence-based feature map for protein function classification. Comput. Biol. Chem..

