# Supplementary material for: DEEPred: Automated Protein Function Prediction with Multi-task Feed-forward Deep Neural Networks
Source: Sci Rep. 2019 May 14;9:7344. doi: 10.1038/s41598-019-43708-3 (PMC6517386; doi:10.1038/s41598-019-43708-3)
Supplement: Supplementary file 1 — Supplementary_Material_1 (Document 1) [file 41598_2019_43708_MOESM1_ESM.docx]

**Supplementary Material 1**

**DEEPred: Automated Protein Function Prediction with Multi-task Feed-forward Deep Neural Networks**

Ahmet Sureyya Rifaioglu^1,2,Ω^, Tunca Doğan^3,4,Ω,*^, Maria Jesus Martin^3^, Rengul Atalay^4^, Volkan Atalay^1,4,*^

^1^ Department of Computer Engineering, METU, Ankara, 06800, Turkey

^2^ Department of Computer Engineering, İskenderun Technical University, Hatay, 31200, Turkey

^3^ European Molecular Biology Laboratory, European Bioinformatics Institute (EMBL-EBI), Hinxton, Cambridge, CB10 1SD, UK

^4^ KanSiL, Department of Health Informatics, Graduate School of Informatics, METU, Ankara, 06800, Turkey

**Ω** Equal contribution

***** Corresponding authors

1. **Literature review on the applications of deep learning in protein function prediction**

Fa et al. developed a GO based protein function prediction method using feed-forward deep neural networks ^1^. The authors generated and tested both single and multi-task feed-forward DNN architectures. The input of the method was 258-dimensional feature vectors covering 14 different functional and structural aspects. The models were trained for 868 different GO terms. Due to computational intensity issues, the authors first extracted branches on GO DAG for each GO category and they trained several multi-task DNN models where each model includes GO terms in a branch on the GO DAG. The results indicated that there was no significant difference between the performances of different DNN architectures. In another study, Liu employed deep recurrent neural networks for automated protein function prediction using amino acid sequences as the input ^2^. Training and prediction were performed for only four protein classes (i.e., families). To generate input vectors, protein sequences were encoded as one-hot vectors, where each residue was represented as 20 bits representing 20 different amino acids. For training, the author selected a maximum sequence length of 333 amino acid residues. The input vectors of the proteins whose sequence length is less than 333 was padded with zeroes to obtain fixed-sized vectors. ProLanGO is another method that employed recurrent neural networks for GO-based function prediction using amino acid sequences ^3^. In ProLanGO, the authors trained only one model using recurrent neural network architecture which incorporates 42,819 GO terms (i.e., all GO terms that has been annotated to at least one of the 523,990 protein sequences in UniProtKB/Swiss-Prot database); however, performance comparison results indicated a low performance for ProLanGO compared to conventional methods. Gligorijevića et al. proposed deepNF, which extracted complex protein features using deep autoencoders and protein-protein interaction networks ^4^. Features extracted with autoencoders were fed to binary classifiers (i.e., SVMs) to predict 783 GO terms for human. In this study, DNNs were only used for extracting complex protein features, not for the functional classification. Similarly, Chicco et al. ^5^ applied deep autoencoder neural networks to predict a large number of GO terms for the genes of *Bos taurus* and *Gallus gallus* species. The input to the models was binary feature vectors, where each dimension represented the presence or absence of a GO annotation for a gene. In another work, deep restricted Boltzmann machines were applied to predict protein functions for four different species ^6^ using an input feature representation similar to the one employed by Chicco et al. They considered the issue as a multi-task learning problem, and all GO terms (i.e., terms that have at least one protein annotation) were incorporated in a single species specific model. Tavanaei *et al.* employed deep convolutional neural networks and used tertiary structures as the input of their method, to extract features from proteins ^7^. The approach was applied to predict five different GO terms by training five single-task models. DeepGO was another convolutional neural network based method, which incorporated protein sequence and protein-protein interaction information at the input level to construct multi-task predictor models, one for each GO category (i.e., 589 MF, 932 BP and 436 CC GO terms) ^8^. Input proteins were represented by 1000 x 128 (i.e., each row corresponded to a residue and columns represented dense embeddings generated by the embedding layer of the DNN) matrices, which were passed to convolutional and pooling layers for feature extraction. The authors also generated knowledge graph embeddings for each protein using protein-protein interaction networks. Both the protein sequence embedding features and graph embedding features were concatenated and connected to fully-connected layers, which were followed by an output node for each GO term. DeepGO is also presented as a web-service. Finally, Szalkai and Grolmusz proposed "SECLAF", a convolutional neural network based architecture, by adopting a multi-tasking approach. Two models were generated, one to predict 983 GO terms (only shallow terms on the GO DAG were considered) and another one to predict 698 UniProt family classes. In this study, protein sequences were encoded as n x 26 sized matrices, where n corresponds to the length of the sequence (the maximum was 2000 residues) and 26 columns corresponds to 20 one hot encoding for 20 different amino acids plus 6 different physicochemical properties for each amino acid. SECLAF also has a web-service^9^. In some of the studies described above, the authors reported extremely high test performances for their methods. This is most probably due to using neither a hold-out test dataset, nor a test set separated from the training instances by a maximum sequence similarity threshold (i.e., using sequence clustering). This often leads to overfitting of the models on the training data and the overestimation of the test performance.

**2. Hyper-parameters of Deep Neural Networks and The Optimization Analysis**

A hyper-parameter is a parameter, the value of which cannot be adjusted during the training step, and thus should be selected beforehand. For this reason, the same machine learning models are trained multiple times using different hyper-parameter values, to select the ones that provided the best predictive performance. The number of hyper-parameters can be huge in deep learning algorithms; therefore, selection of best hyper-parameters is a challenge. It is known that the success of any hyper-parameter selection is dependent on the data and the architecture of the system ^10^. Hence, optimal hyper-parameters should be searched for each model to be trained, individually. Below we discussed different types of hyper-parameters that we tested for DEEPred.

The most basic hyper-parameters are the number of hidden layers and the number of neurons at each of these layers. Generally, the system performance increases with the increasing number of hidden layers and the number of neurons in these layers, until it saturates at some point. However, the main disadvantage behind using excessive number of layers and neurons is the computational burden. There is a tradeoff between computational complexity, which can easily render these models impractical to run, and the predictive performance. Finding the optimal point is a challenging task and an active area of research. The strategy currently followed in the literature is testing a large number of parameter values, which was also assumed in this study.

Deep learning algorithms generally suffer from the problem of overfitting, where predictive models may perform well on training data but not on test data. Several approaches were proposed to avoid overfitting during the training of deep neural networks, known as the regularization techniques ^11–13^. One of the most popular regularization techniques is the dropout method ^12^. Dropout method randomly removes some of the neurons from different layers along with their connections at every iteration during the training procedure, so that the system is directed to find a more generalized state, that is not dependent on a few neurons and connections. Another widely used regularization technique is the input normalization, where input features are normalized to zero mean and a variance of one. Batch normalization is another method that was proposed to reduce the effect of parameter initialization, to speed up the training and to reduce overfitting. Batch normalization is similar to input normalization; however, the aim here is the normalization of inputs of each hidden layer instead of the normalization of the input feature vectors ^14^.

Optimization algorithms are used to minimize an objective function, which includes learnable parameters (i.e., weights and biases) of a deep learning system. The learnable parameters are updated at each iteration using the optimization algorithm so that the system converges to an optimal solution. Several optimization algorithms were proposed in recent years, each containing one or more hyper-parameters ^10^. Most widely used optimization algorithms are ADAM, RMSProp and Momentum ^15,16^. A critical hyper-parameter that is related to the optimizer is the learning rate. Briefly, learning rate value decides how much the weights should be changed (in the direction of the gradient) at each iteration. If it is selected to be very low, the training would be more reliable; however, the training process takes longer time. If it is selected to be very high, the training would be fast but unreliable (i.e., produces low performance models). Finding the optimal point for the learning rate is critical. There are also other optimizer dependent hyper-parameters such as momentum (Momentum), beta1-beta2 (Adam) and decay (RMSProp). In this study, we used default hyper-parameters for these optimizers.

In DEEPred, the total number of model-training-runs was huge (i.e., more than 100,000) due to the high number of selected hyper-parameter value combinations. In order to select the hyper-parameter values, we trained three sets of GO terms, chosen from different levels of GO hierarchy, with varying number of protein associations. The aim of selecting the GO terms in this way was to come up with a small GO term set, which can represent the whole system, since employing all GO terms in this test was not possible due to extremely high computational complexity. Based on the predictive performance results of different runs, we reduced the number of hyper-parameters into a smaller set of options for the training of the whole system. We used TensorFlow framework for training the models and all computations were distributed on 2500 CPU cores in our supercomputing cluster ^17^. The statistics and results of the hyper-parameter optimization tests are explained in the Results section.

**3. Performance Evaluation Metrics**

Recall, precision, F-max and Smin measures, which are given in the Equation 6, Equation 7, Equation 8 and Equation 9; are used to evaluate the performance of the system. *TP, FP, TN* and *FN* represents the number of true positives, false positives, true negatives and false negatives; respectively.

$${Recall}_{\tau i}=\frac{{TP}_{\tau i}}{{TP}_{\tau i}+{FN}_{\tau i}}$$

(6)

$${Precision}_{\tau i}=\frac{{TP}_{\tau i}}{{TP}_{\tau i}+{FP}_{\tau i}}$$

(7)

$$F_{max}=\max_{i=1\ldots N} \left\{ \frac{{2*Pr}_{\tau i}*{Rc}_{\tau i}}{{Pr}_{\tau i}+{Rc}_{\tau i}} \right\}$$

(8)

$$S_{min}=\min_{i=1\ldots N} \left\{ \sqrt{{{Ru}_{\tau i}}^{2}{{+ Mi}_{\tau i}}^{2}} \right\}$$

(9)

In equations 6, 7, 8 and 9; *τ_i_* represent the *i*^th^ probabilistic score threshold. Fmax correspond to the maximum of the F1-score values (described inside the curly brackets in Equation 8), calculated for each arbitrarily selected probabilistic score threshold. *i*=1*...N* represents there are *N* different arbitrarily selected probabilistic score thresholds. *Ru_τi_* and *Mi_τi_* in Equation 9 corresponds to remaining uncertainty and normalized misinformation, respectively. Smin is the minimum semantic distance.

For CAFA2 and CAFA3 benchmark tests, we calculated the F-max scores in exactly the same way as it was described in CAFA2 GitHub repository ^18^. Performance evaluation scripts released by CAFA team were directly used for this purpose. Additional information regarding CAFA performance measures and scripts can be obtained from Jiang *et al.* ^19^.

**4. Protein Feature Types and Vector Generation**

Conjoint triad feature ^20^ considers the frequencies of amino acid triplets (i.e., consecutive three residues on the sequence). Here, query protein sequences are encoded by the frequency of the occurrence of each triplet combination. Since the total number of combinations are quite high (i.e., 20x20x20 = 8,000), Conjoint triad considers reduced alphabet by using amino acid groups generated by considering their physicochemical properties. This way, each protein is represented as a 343-dimensional (i.e., 7x7x7) feature vector. There exist several studies in the literature that employ the conjoint triad feature ^21–24^.

Pseudo-amino acid composition (PAAC) feature ^25^ incorporates single amino acid frequency information (i.e., conventional amino acid composition) together with sequence correlation factors without losing the sequence-order information in a protein sequence. This method computes a set of coupling factors using the physicochemical properties of amino acids (i.e., hydrophobicity, hydrophilicity value and the side chain mass) and records them in a 50-dimensional descriptor vector in an ordered fashion. PAAC feature has frequently been employed in the literature ^26–30^.

Subsequence profile map (SPMap) is a method for functional classification of protein sequences, based on the extraction and clustering of short sub-sequence features ^31^. Here we only incorporated the sub-sequence based feature vector generation module of the SPMap method. For this, all fixed-length subsequences are extracted from the protein sequences and the extracted subsequences are grouped using a hierarchical clustering approach, based on BLOSUM-62 matrix. Finally, obtained clusters are transformed into probabilistic profiles and protein sequences are converted into feature vectors based on the distribution of their sub-sequences over the generated probabilistic profiles. The original SPMap method constructs a profile for each GO term (i.e., for each model) individually. This results in protein feature vectors with varying sizes. In this study, we modified the SPMap algorithm to generate a single reference probabilistic profile using all protein sequences in the training dataset associated with all GO terms in a specific GO category. Therefore, each protein sequence was represented by a fixed-dimensional feature vector for all models, resulting in 1893, 1861 and 1901-dimensional vectors for MF, BP and CC categories, respectively. Conjoint triad and pseudo-amino acid composition features were extracted from protein sequences using the ProtR software ^32^. SPMap features were calculated using our in-house software. For all methods, we used default parameters to generate the feature vectors.

**5. Predictive Performance Evaluation Datasets**

The hold-out validation aims to determine and fine tune the hyper-parameter values and to observe the performance of the system. The hold-out datasets were constructed as follows: the training dataset for each GO term (see the "Training Dataset Construction" section) was randomly divided into two datasets such that 80% of the annotations were reserved for the training and 20% of the samples were used for the hold-out validation dataset. The proteins in the validation dataset were fed to the trained models to produce GO term predictions. We then compared the resulting predictions with the true annotations of these proteins to calculate the performance metrics, which are explained in the next section.

We used CAFA2 challenge benchmark dataset for the independent performance evaluation and for the comparison with the state of the art methods (i.e., the methods participated to the CAFA2 challenge). Since there is a temporal difference between CAFA2 challenge and the date we trained our system, (to yield a fair comparison) we had to remove the training instances (i.e., annotations) that were released in UniProt-GOA after the CAFA2 participation deadline, from our training dataset, and re-train our system. We then directly fed CAFA2 challenge benchmark dataset proteins as query instances to our trained models (the annotations in the CAFA2 benchmark dataset did not overlap with our training datasets). The CAFA2 benchmark dataset contained 1,828 proteins, 997 GO terms and 3,187 annotations for MF; 2,618 proteins, 3,375 GO terms and 7,956 annotations for BP; 2,938 proteins, 587 GO terms and 5,085 annotations for CC category of GO. The statistics of the CAFA3 benchmark dataset are given in the main text, Methods section 2.5.

**6. DEEPred Hyper-parameter Optimization Results**

This analysis contained nearly 100,000 model runs with varying parameters; as a result, we decided provide a summary table (Table S.1). The detailed result tables of the large-scale hyper-parameter tests are given in Supplementary Material 3. In Table S.1, the average performance result for each hyper-parameter selection is shown. For example, the performance value given for the dropout rate 0.6 is the average performance of all the tests, where the dropout rate was kept constant at 0.6 and the rest of the parameters changed across the given ranges in Table S.1. This way, different values of the same hyper-parameter became comparable to each other. Selected hyper-parameter values at the end of this performance test are highlighted with bold font in Table S.1. For some of the hyper-parameters, more than one value has been selected. This means that, all of these selected values were used during the training of the whole system, and the value that provide best training performance was finally selected for the corresponding model. For all models, large-scale hyper-parameter optimization test served as a preliminary elimination analysis to reduce the training run times. As observed from Table S.1, the average performances were close to each other in most cases. This was mainly due to selecting hyper-parameter values that are frequently employed in the DNN literature. The reason behind selecting 2 hidden layers instead of 3 was that, the observed performance gain was not sufficient to compensate for the increased computational run times. The learning rate 0.01 was selected among 0.001 and 0.0005 even though it produced an inferior average performance; however, in some of the models 0.01 produced significantly better results compared to the other values. Considering the number of neurons at each hidden layer, values such as (600,400), (2200,600), (200,25) etc. were selected as pairs for the first and second hidden layers, respectively.

**Table S.1:** Hyper-parameter names, their ranges used in this study and the optimization test performance results.

| **Hyper-parameter Name** | **Range** | **Average Model Perf. (F1-score)** |
| --- | --- | --- |
| Input Normalization | **Yes** | 0.50 |
|  | No | 0.50 |
| Learning rate | **0.0005** | 0.53 |
|  | **0.001** | 0.52 |
|  | **0.01** | 0.47 |
|  | 0.1 | 0.43 |
| Number of hidden layers | **2** | 0.50 |
|  | 3 | 0.51 |
| Number of neurons at each layer | 100, 200, 400, 1000, 2000, … | * |
| Optimizer | **Adam (default)** | 0.51 |
|  | Momentum (default) | 0.48 |
|  | **RMSprop (default)** | 0.52 |
| Mini-batch size | **32** | 0.51 |
|  | 64 | 0.50 |
| Drop-out rate | 0.6 | 0.49 |
|  | **0.8** | 0.51 |
| Batch Normalization | **Yes** | 0.53 |
|  | No | 0.47 |
| * For each model, the number of neurons at each hidden layer is variable, and most of the possible combinations are tested (e.g., for models with 2 hidden layers: 100 & 200, 100 & 400, 200 & 400 and etc.) Since the number of neurons at different hidden layers should be considered in combination, it was not feasible to add all results into this table. The detailed result for different combinations of hidden units can be observed in Supplementary Material 3. | | |

**7. Effect of the GO Term levels on the System Performance**

The aim in this test was to observe if there is any relation between specificity of the GO terms on the GO DAG and the prediction performance. In our previous work, we found out that shallow/generic GO terms (i.e., the ones with low levels) are difficult to predict due to the heterogeneity of the sequence signatures caused by the high number of annotated sequences from diverse protein families ^33^. Here, we aimed to observe the case when DNNs are employed. Each plot in Figure S.1 displays the F1-score performances of the models (i.e., each colored dashed line) that contain GO terms from distinct levels, for each GO category. For example, blue dashed line in the first plot of Figure S.1 represents the performance of the DNN model that incorporates the GO terms from the GO level 1, and the horizontal axis further divides these performance measures to GO terms with varying number of training instances. We still chose to include the training dataset size separation in Figure S.1 in order to observe the mutual effect of GO levels and dataset sizes on the performance. As observed from Figure S.1, F1-score performances are highly variable for each GO level (e.g., values varied between 0.24 and 0.88 for different levels in the MF category); and that, there is no correlation between GO levels and the performance of the system. These results indicated that DNN-based models do not perform worse in low levels (i.e., on shallow/generic GO terms), on the contrary, they perform sufficiently well, as long as high number of training instances are provided (which usually is granted for shallow terms). Also, the performance for high level GO terms (i.e., specific terms) are not low, as well. It can be said that, the only main factor driving the performance here is the number of training instances.


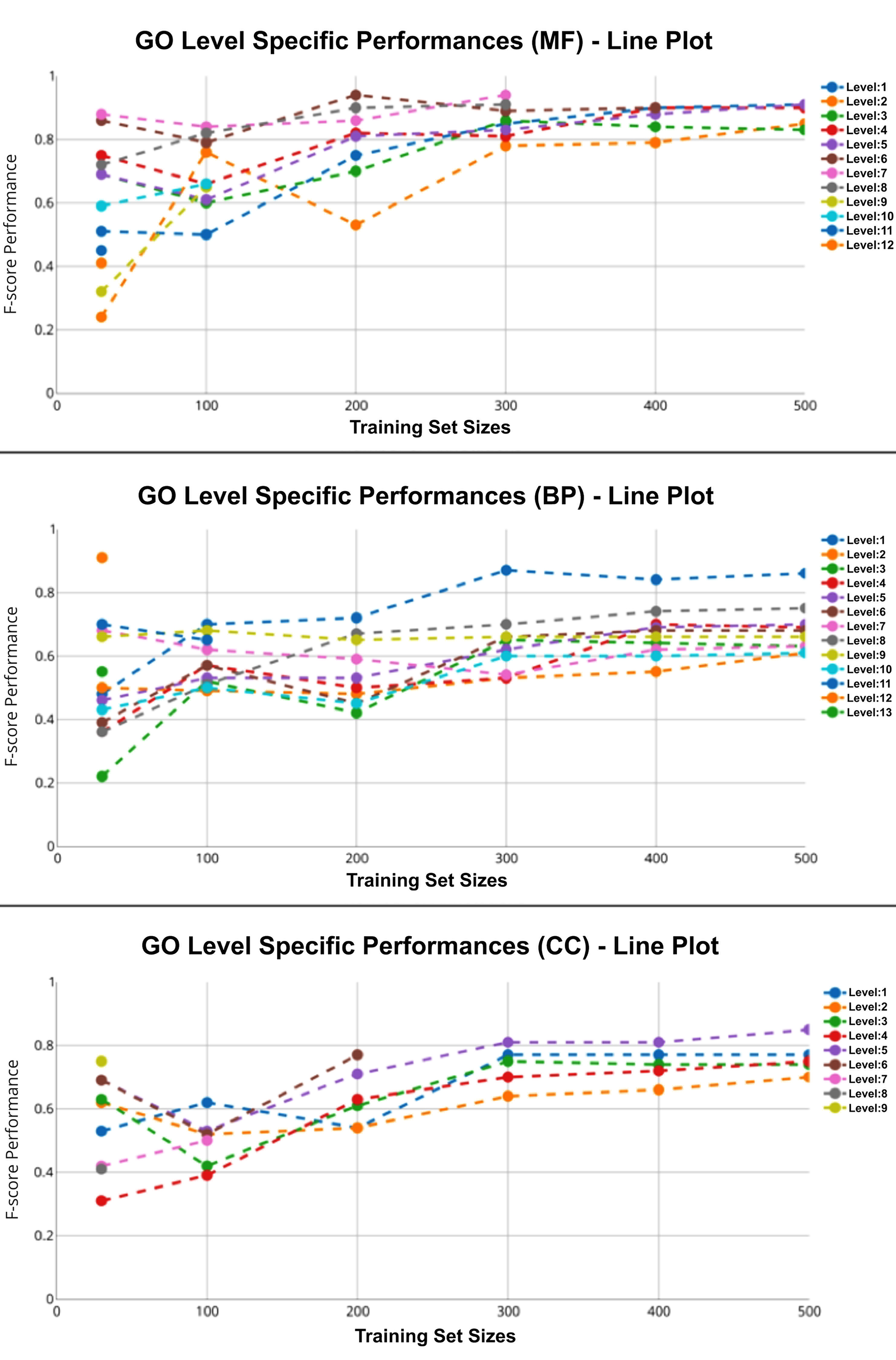


**Fig S.1**: Line plots displaying GO level specific performance in F1-score values (vertical axis) for six training datasets (horizontal axis) for each GO category. In this analysis, the training was done using only the annotations with manual experimental evidence codes.

**8. An introduction about *P. aureginosa* and the biofilm formation**

*Pseudomonas aureginosa* is a gram-negative pathogenic bacteria with high medical importance due to its ability to cause infection in human (e.g., pneumonia) and its highly effective antibiotic resistance mechanisms ^34^. An important factor contributing to the infectious capabilities of various bacterial and fungal species is their ability to form biofilms. A biofilm is a matrix layer made up of extracellular polymers and the microorganisms themselves. Biofilms adhere to solid surfaces and provide a medium for the cells to proliferate and resistance to environmental stress. Due to this reason, understanding the biofilm formation mechanisms in pathogenic microorganisms have high importance ^35,36^.

**References**

1. Fa, R., Cozzetto, D., Wan, C. & Jones, D. T. Predicting Human Protein Function with Multi-task Deep Neural Networks. *bioRxiv* ***256420*,** (2018).

2. Liu, X. L. Deep Recurrent Neural Network for Protein Function Prediction from Sequence. *arXiv* 1–38 (2017).

3. Cao, R. *et al.* ProLanGO: Protein function prediction using neural machine translation based on a recurrent neural network. *Molecules* **22,** (2017).

4. Gligorijević, V., Barot, M. & Bonneau, R. deepNF: Deep network fusion for protein function prediction. *bioRxiv* **223339** (2017).

5. Chicco, D., Sadowski, P. & Baldi, P. Deep autoencoder neural networks for gene ontology annotation predictions. *Proc. 5th ACM Conf. Bioinformatics, Comput. Biol. Heal. Informatics - BCB ’14* 533–540 (2014).

6. Zou, Xianchun; Wang, G. & Guoxian. Protein Function Prediction Using Deep Restricted Boltzmann Machines. *BioMed Res. Int. Vol.* **2017,** 1–9 (2017).

7. Tavanaei, A. *et al.* Towards Recognition of Protein Function based on its Structure using Deep Convolutional Networks. *IEEE Int. Conf. Bioinforma. Biomed.* 145–149 (2016).

8. Kulmanov, M., Khan, M. A. & Hoehndorf, R. DeepGO: Predicting protein functions from sequence and interactions using a deep ontology-aware classifier. *Bioinformatics* **34,** 660–668 (2017).

9. Szalkai, B., Grolmusz, V. & Hancock, J. SECLAF: A Webserver and Deep Neural Network Design Tool for Hierarchical Biological Sequence Classification. *Bioinformatics* **34,** 2487–2489 (2018).

10. Angermueller, C. *et al.* Deep Learning for Computational Biology. *Mol. Syst. Biol.* **12,** 1–16 (2016).

11. Hinton, G. E., Osindero, S. & Teh, Y. W. A fast learning algorithm for deep belief nets. *Neural Comput.* **18,** 1527–54 (2006).

12. Srivastava, N., Hinton, G., Krizhevsky, A., Sutskever, I. & Salakhutdinov, R. Dropout: A Simple Way to Prevent Neural Networks from Overfitting. *J. Mach. Learn. Res.* **15,** 1929–1958 (2014).

13. Goodfellow, I., Bengio, Y. & Courville, A. *Deep Learning*. (MIT Press, 2016).

14. Windows, M. *et al.* Batch Normalization: Accelerating Deep Network Training by Reducing Internal Covariate Shift. *arXiv* **1011.1669v,** 1–9 (2014).

15. Kingma, D. P. & Ba, J. Adam: A Method for Stochastic Optimization. *arXiv* 1–15 (2014).

16. Sutskever, I., Martens, J., Dahl, G. & Hinton, G. On the importance of initialization and momentum in deep learning. *Proc. Mach. Learn. Res.* **28,** 1139–1147 (2013).

17. Abadi, M. *et al.* TensorFlow: Large-Scale Machine Learning on Heterogeneous Distributed Systems. *ArXiv* **1,** 1–19 (2015).

18. CAFA2 GitHub Repository. Available at: https://github.com/yuxjiang/CAFA2. (Accessed: 14th August 2017)

19. Jiang, Y. *et al.* An expanded evaluation of protein function prediction methods shows an improvement in accuracy. *Genome Biol.* **17,** 1–19 (2016).

20. Shen, J. *et al.* Predicting protein-protein interactions based only on sequences information. *Proc. Natl. Acad. Sci. U. S. A.* **104,** 4337–41 (2007).

21. Wang, Y.-C., Wang, Y., Yang, Z.-X. & Deng, N.-Y. Support vector machine prediction of enzyme function with conjoint triad feature and hierarchical context. *BMC Syst. Biol.* **5,** S6 (2011).

22. Wang, Y.-C., Wang, X.-B., Yang, Z.-X. & Deng, N.-Y. Prediction of enzyme subfamily class via pseudo amino acid composition by incorporating the conjoint triad feature. *Protein Pept. Lett.* **17,** 1441–1449 (2010).

23. You, Z.-H., Chan, K. C. C. & Hu, P. Predicting Protein-Protein Interactions from Primary Protein Sequences Using a Novel Multi-Scale Local Feature Representation Scheme and the Random Forest. *PLoS One* **10,** e0125811 (2015).

24. Guo, Y., Yu, L., Wen, Z. & Li, M. Using support vector machine combined with auto covariance to predict protein-protein interactions from protein sequences. *Nucleic Acids Res.* **36,** 3025–3030 (2008).

25. Chou, K.-C. Prediction of Protein Cellular Attributes Using Pseudo- Amino Acid Composition. *Proteins Struct., Funct., Genet.* **255,** 246–255 (2001).

26. Qiu, W. R., Xiao, X., Lin, W. Z. & Chou, K. C. IMethyl-PseAAC: Identification of protein methylation sites via a pseudo amino acid composition approach. *Biomed Res. Int.* **2014,** (2014).

27. Xu, Y. *et al.* INitro-Tyr: Prediction of nitrotyrosine sites in proteins with general pseudo amino acid composition. *PLoS One* **9,** (2014).

28. Liu, B., Wang, S. & Wang, X. DNA binding protein identification by combining pseudo amino acid composition and profile-based protein representation. *Sci. Rep.* **5,** 1–11 (2015).

29. Limongelli, I., Marini, S. & Bellazzi, R. PaPI: pseudo amino acid composition to score human protein-coding variants. *BMC Bioinformatics* **16,** 123 (2015).

30. Chou, K. C. Using amphiphilic pseudo amino acid composition to predict enzyme subfamily classes. *Bioinformatics* **21,** 10–19 (2005).

31. Sarac, O. S., Gürsoy-Yüzügüllü, O., Cetin-Atalay, R. & Atalay, V. Subsequence-based feature map for protein function classification. *Comput. Biol. Chem.* **32,** 122–30 (2008).

32. Xiao, N., Cao, D. S., Zhu, M. F. & Xu, Q. S. Protr/ProtrWeb: R package and web server for generating various numerical representation schemes of protein sequences. *Bioinformatics* **31,** 1857–1859 (2015).

33. Doğan, T. *et al.* UniProt-DAAC: domain architecture alignment and classification, a new method for automatic functional annotation in UniProtKB. *Bioinformatics* **32,** 2264–2271 (2016).

34. Stover, C. K. *et al.* Complete genome sequence of Pseudomonas aeruginosa PAO1, an opportunistic pathogen. *Nature* **406,** 959–964 (2000).

35. Costerton, J. W., PS, S. & EP, G. Bacterial Biofilms: A common cause of persistent infections. *Science (80-. ).* **284,** 1318–1323 (1999).

36. Donlan, R. M. & Costerton, J. W. Biofilms: survivalmechanisms of clinically relevant microorganisms. *Clin.Microbiol. Rev.* **15,** 167–19 (2002).
